# Supplementary material for: Coupling chromosome organization to genome segregation in Archaea
Source: Nat Commun. 2025 Jul 22;16:6759. doi: 10.1038/s41467-025-61997-3 (PMC12284271; doi:10.1038/s41467-025-61997-3)
Supplement: Supplementary file 1 — Supplementary Information [file 41467_2025_61997_MOESM1_ESM.pdf]

Supplementary Information

Coupling chromosome organization to genome segregation in Archaea

Azhar F. Kabli[1], Irene W. Ng[1], Nicholas Read[1], Parul Pal[2], Julia Reimann[3], Ngat T. Tran[2], Sonja-Verena Albers[3], Tung B. K. Le[2] and Daniela Barillà[1*]

[1]Department of Biology, University of York, York YO10 5DD, United Kingdom

[2]Department of Molecular Microbiology, John Innes Centre, Norwich, United Kingdom

[3]Molecular Biology of Archaea, Microbiology, Faculty of Biology, University of Freiburg, SchänzlestraBe 1, 79104 Freiburg, Germany

[*]Corresponding author: Daniela Barillà, daniela.barilla@york.ac.uk

Supplementary Figures

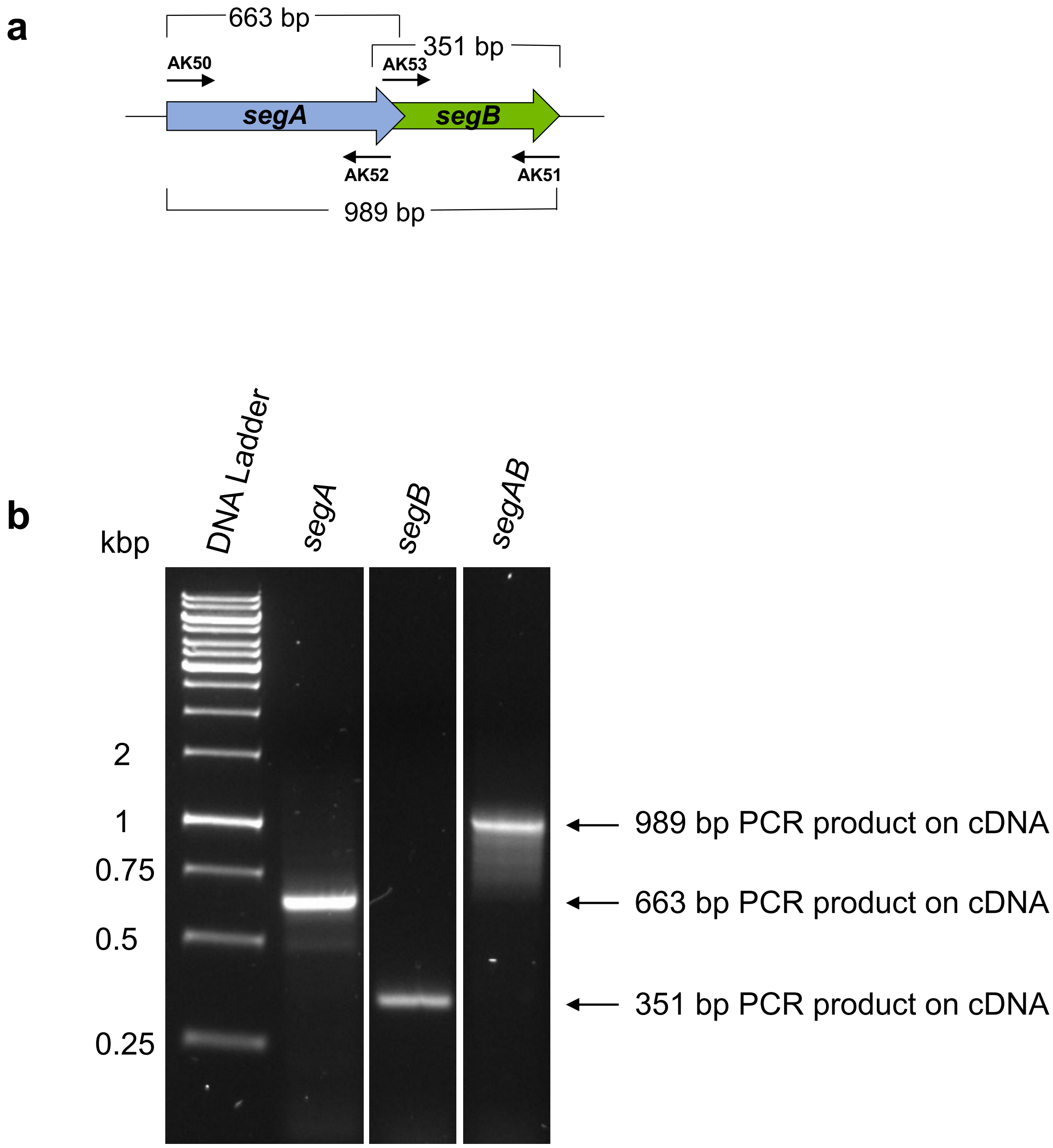

Supplementary Fig. 1. The *segAB* genes form an operon. a Schematic of the *segAB* locus in *S. solfataricus*. Primers used to amplify fragments are indicated (not to scale) and the predicted amplicon sizes are shown. **b** PCR products obtained using cDNA as template and analysed on a 1% agarose gel stained with Syber Safe. The uncropped gel is included in the Source Data file.

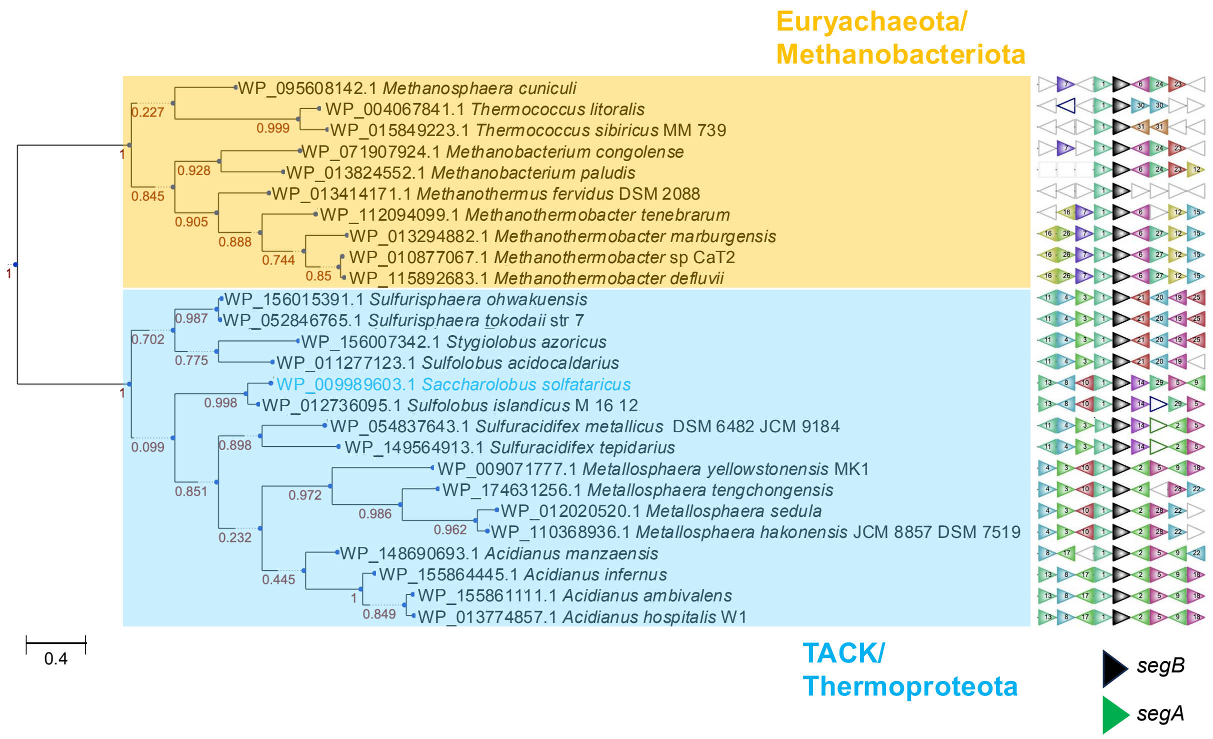

Supplementary Fig. 2. Phylogenetic tree of SegB orthologues among archaea. The tree was constructed using the FlaGs programme[1] that deploys the tree-building feature of ETE v3 Python environment[2]. Bootstrap values are shown next to the branches. The two major clades are highlighted in yellow-orange (Euryarchaeota/Methanobacteriota) and blue (TACK/Thermoproteota). The genomic neighbours of each *segB* orthologue are shown on the diagram on the right side, in which the arrows represent genes that are coloured and numbered as in Figure 1. The *segB* gene is shown in black and *segA* gene in green.

Metallosphaera hakonensis DSM7519 **(300 aa) (12.5 repeats)**

```
MSEIDFLLKRKKKNEGVQGGESGAQRVESRVQGGESGAQRVESRVQGGESGAQRVESRVQGGESGAQRVESRVQG
GESGAQRVESRVQGGESGAQRVESRVQGGESGAQRVESRVQGGESGAQRVESRVQGGESGAQRVESRVQGGESGA
QRVESRVQGGESGAQRVESRVQGGESGAQRVESRVQGGESGAQTTIDITLDSTGIVRTPESRAVSYVGTGIAQES
SSVNLDLMENFLDKDPKIGVWSYPSYIVLQYLYHVKPGFKMSKIAKEALELGMRQLFPDLYSKAEELSRKKGLLK
```

Metallosphaera sedula **(272 aa) (11 repeats)**

```
MSELDFLLKRKKKTEGVQGGESGGQRVESRVQGGESGGQRVESRVQGGESGGQRVESRVQGGESGGQRVESRVQG
GESGGQRVESRVQGGESGGQRVESRVQGGESGGQRVESRVQGGESGGQRVESRVQGGESGGQRVESRVQGGESGG
QRVESRVQGGESGGQRVESIQISPETDSHQNGNTEQTTQTVTQTSVDQTSLEQLMESLLDKDPKIGVWSYPGYAV
LQYLYHTKPGFRMSKVAKDALELGLKQLYPDLYSKAESLARQKGLIK
```

Metallosphaera tengchongensis **(247 aa) (8.5 repeats)**

```
MSELDFLLKRKKKDEQKSGESVEKRTEGRVQSGESVEKRTEGRVQSGESVEKRTEGRVQSGESVEKRTEGRVQSG
ESVEKRTEGRVQSGESVEKRTEGRVQSGESVEKRTEGRVQSGESVEKRTEGRVQSGESVERPNIHEVELNVVTIG
NVKPDLPPRDDAQENTETPSSGIDEVEKLMGNFLDKDPKIGVWSYPSYMVLQYLFHTKPGFKMSKMAKDALEIGM
RQLFPDLYSKAEKIAKDKGLLR
```

Metallosphaera cuprina **(269 aa) (11 repeats)**

```
MSELDFLLNRKKRDGGVQGVDNSPQRVDNRVQGVDNSPQRVDNRVQGVDNSPQRVDNRVQGVDNSPQRVDNRVQG
VDNSPQRVDNRVQGVDNSPQRVDNRVQGVDNSPQRVDNRVQGVDNSPQRVDNRVQGVDNSPQRVDNRVQGVDNSP
QRVDNRVQGVDNSPQRVDNTIDSGADKSPMISLENQNVKRDQRLEDEELELMRKFLDKDPKIGIWSYPSYVVLQY
LFNTRPGFKISKVAKEALEIGMRQLFPDLYTKAELITKQRGLVK
```

Metallosphaera yellowstonensis **MK1 (172 aa) (no repeats)**

```
MSELDFLLNRKKSVGKTSDSRVEKEEEIGLESRENRGEALPQTTESSGKSVEERGKPEESSGLTLESLGKSVEGT
PEVEMETLEERGNPLQSGVSRDKRSIESVMMTLLSREPKIGVWSYPSFLVLQYLFSTKPGFRMSKIAKEALEIGL
RQLYPELFAIAESVAKEKGLIK
```

Methanobacterium congolense **(168 aa) (no repeats)**

```
MSQKKKRESALGRGLDALIRTQPVEEPGEKEQDVESEIPAEEEKPTQRKTPPAKKTRKPSKTSGRSTAKTARKSP
EKAQKPKIPRPKKKPETAAEDFNVDSQLLEEVMAEVAKNPRISLWSAKSAAVLRYLRKTKPAFSISKEASALIED
AVKEKYPDLWEIFEGEGL
```

Acidianus manzaensis **(170 aa) (no repeats)**

```
MSELDFLLNRKKNLEKKDKKEESREETKTNTEEKIQNTQEQIEKSEERKTEETKIQNIDSIENSSNSIVMKEESA
ENNQYSREEKTENSRVENAEDREDNVESIMKTFLNKDPKIGVWSYPSFLVLQYLYNTKPGFKMSKVAKDALEYGL
KRMYPELFDKAEKISQSKIR
```

Sulfolobus acidocaldarius **(151 aa) (2 short repeats)**

```
MSELDLILNRKKTEQKVEASEQKVESRQETSKSTTQKLAETKTSETQEKVNENDASKSPREDLEEVKSSPNSNSE
VKVDNIKGIMKKFIDRDPKIGIWSYPSFLVLQYLYHTVPGFKMSKIAKDALERGLREIYPDLFRIAEEVTLESKK
S
```

Saccharolobus solfataricus **(109 aa) (no repeats)**

```
MSELDFLLKKKRKSEDEEKIINNNENAKKEEITNEEEKIKNDMLKYIEKDPKIGVWSYPAFLVLQYLYHTVPGFK
MSRTAKEALEKGLKEMYPTLFTIAEKIAKERFKE
```

Supplementary Fig. 3. SegB orthologues harbour repeat sequences. Amino acid sequences of SegB proteins in different archaeal genera. The repeats are highlighted in colour.

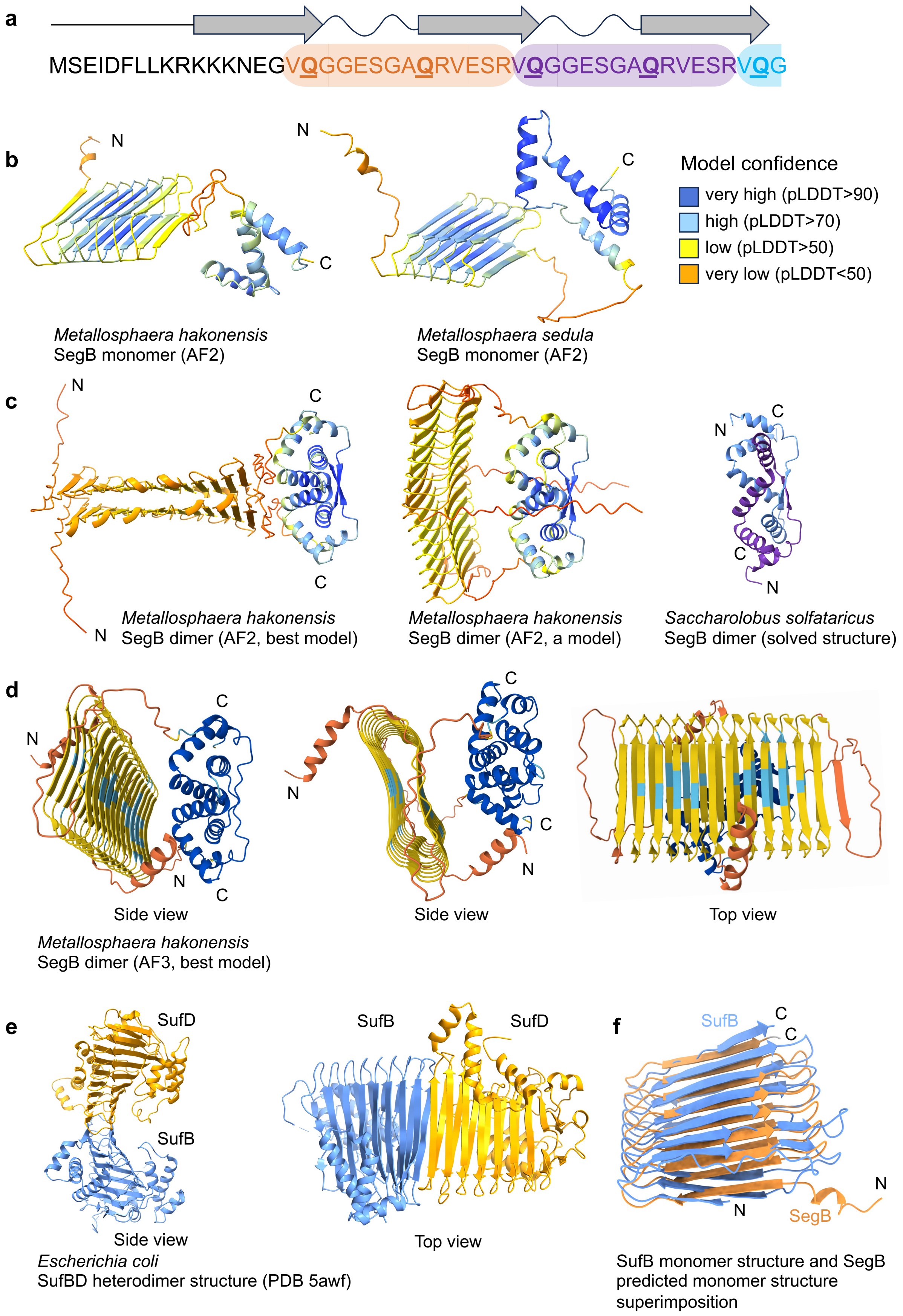

Supplementary Fig. 4. SegB from *Metallosphaera hakonensis* contains repeats and has a predicted β-helix structure domain. a SegB protein sequence in which the repeats are highlighted in coloured boxes and the predicted secondary structure is shown above. Each

repeat starts at the end of a β-strand and extends until the next β-strand, including the intervening loop. **b** AlphaFold2-predicted structures of *Metallosphaera hakonensis* (Supplementary Data 1) and *Metallosphaera sedula* SegB monomer (Supplementary Data 2) coloured according to level of confidence. **c** AlphaFold2-predicted structures of *Metallosphaera hakonensis* SegB dimer (*left*, AF2 proposed best model; *middle*, one of the predicted models) (Supplementary Data 3 and 4) and experimentally determined X-ray structure of *S. solfataricus* SegB dimer (PDB 7dv2) (*right*). **d** AlphaFold3-predicted structure of *Metallosphaera hakonensis* dimer coloured according to the level of confidence (Supplementary Data 5). **e** Structure of *E. coli* SufBD heterodimer (PDB 5awf)[3] (*left*, side view; *middle*, top view). SufB is shown in blue and SufD in yellow-orange. **f** Superimposition of SufB (blue) and AF2-predicted SegB (orange) β-helix domains.

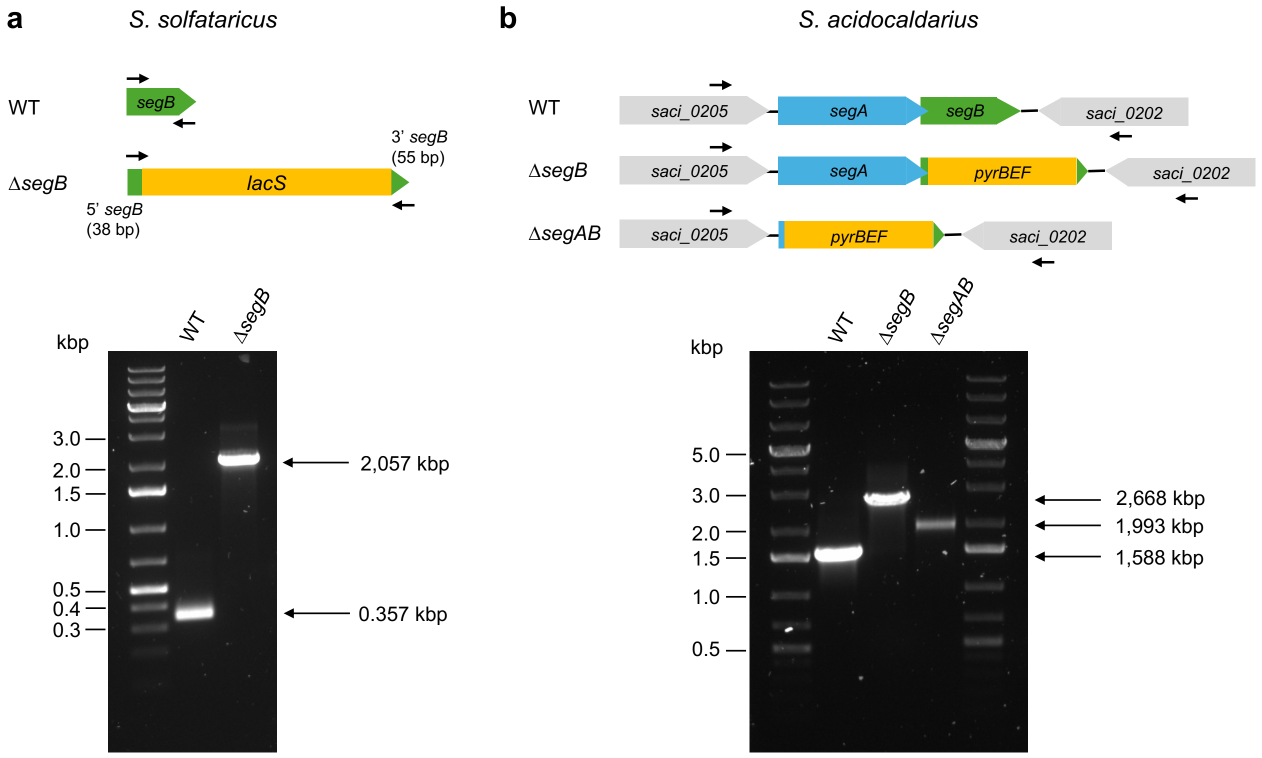

Supplementary Fig. 5. Verification of *S. solfataricus* and *S. acidocaldarius* deletion strain by PCR. a Schematic of the *S. solfataricus* wild type and deleted *segB* gene that was constructed by replacement with the *lacS* gene. The primers used for the diagnostic PCRs are shown as black arrows (not to scale) (*top*). 1% agarose gel showing the PCR products obtained by amplifying the gene using genomic DNA as template (*bottom*). **b** Schematic of the *S. acidocaldarius* genomic region containing the *segAB* genes in the wild type strain and of the same region in the deletion strains in which *segB* or *segAB* are replaced by *pyrBEF* genes. The primers used for the diagnostic PCRs are shown as black arrows (not to scale) (*top*). 0.8% agarose gel showing the PCR products obtained by amplifying the genes using genomic DNA as template (*bottom*).
Uncropped images of the gels are provided in the Source Data file.

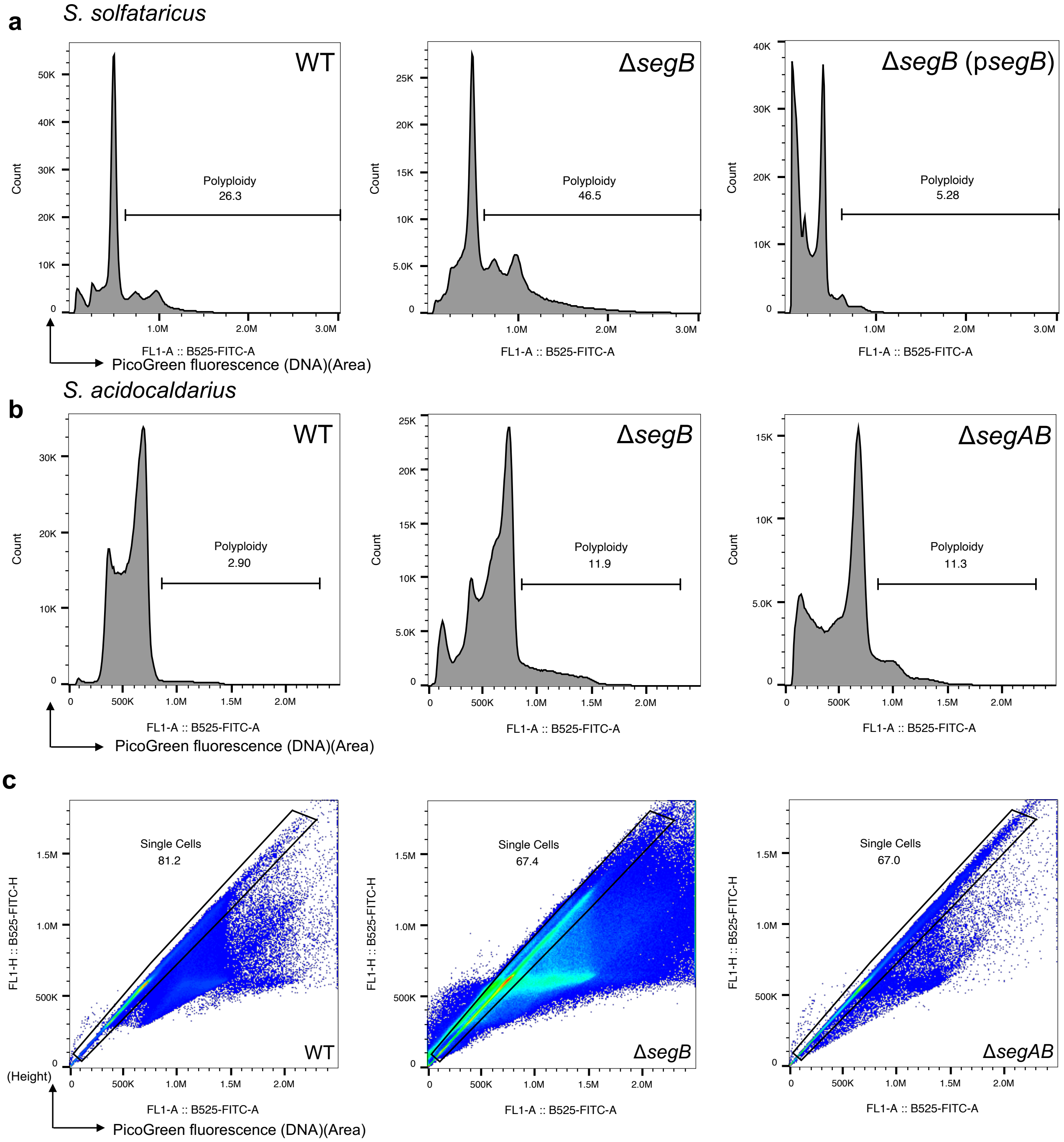

Supplementary Fig. 6. Δ*segB* and Δ*segAB* deletion mutants are enriched in polyploid cells. Representative flow cytometry histograms of wild type and deletion strains highlighting the percentage of polyploid cells in *S. solfataricus* (**a**) and *S. acidocaldarius* (**b**). The X axis indicates the fluorescence area and the Y axis shows the cell count. The chromosomal DNA was stained with PicoGreen fluorescent dye. Either two or three independent biological samples were used for each strain. The polyploidy mean ± SD are: *S. solfataricus* (wild type, PBL2025 = 26.27 ± 0.45; Δ*segB* = 45.20 ± 1.83; complemented Δ*segB* (p*segB*) = 3.43 ± 1.73); *S. acidocaldarius* (wild type, MW001 = 2.88 ± 0.005; Δ*segB* = 10.71 ± 1.92; Δ*segAB* = 8.23 ± 2.69). Source data are provided as Source Data file. For each repeat ~ 10^6 cells were analyzed. **c** Gating strategies adopted to isolate single cells and used to derive the *S. acidocaldarius* histograms shown in panel B. The percentage of single cells within the gates is indicated in each plot. The X axis indicates the PicoGreen area and the Y axis indicates the height.

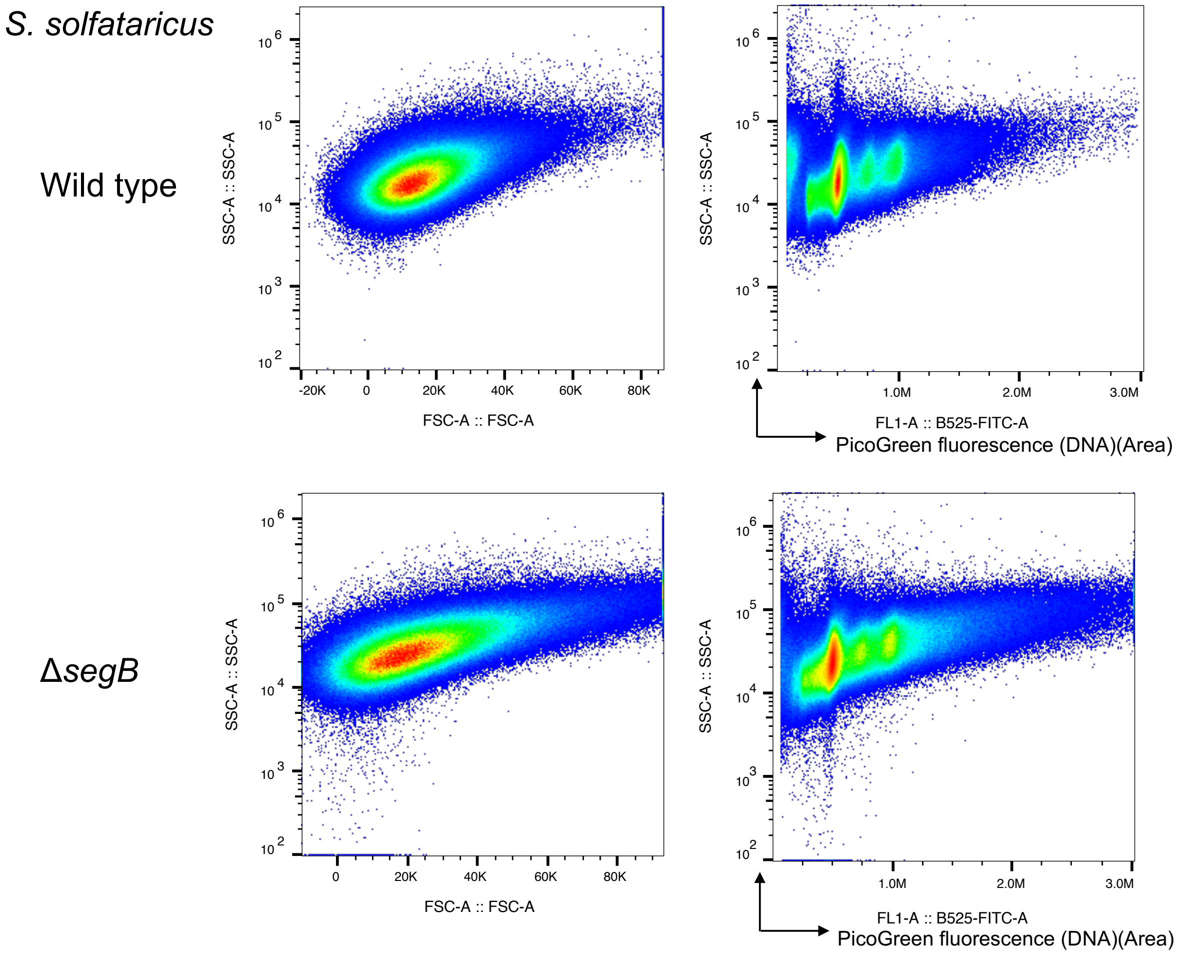

Supplementary Fig. 7. *S. solfataricus* Δ*segB* shows a more heterogeneous population with larger polyploid cells that exhibit higher complexity compared to the wild type strain. Flow cytometry dot plots of *S. solfataricus* wild type and Δ*segB* cells displaying the Forward Scatter values on the X axis and the Side Scatter values on the Y axis (*left*) or the PicoGreen fluorescence values *versus* the Side Scatter values (*right*). The Forward Scatter is reflective size and Side Scatter reflects the internal organization and complexity of the cells. Each dot represents a cell and approximately 700.000 single cells are displayed in the plots. The colour indicates the density of the cells, blue correlates with low and red with high density.

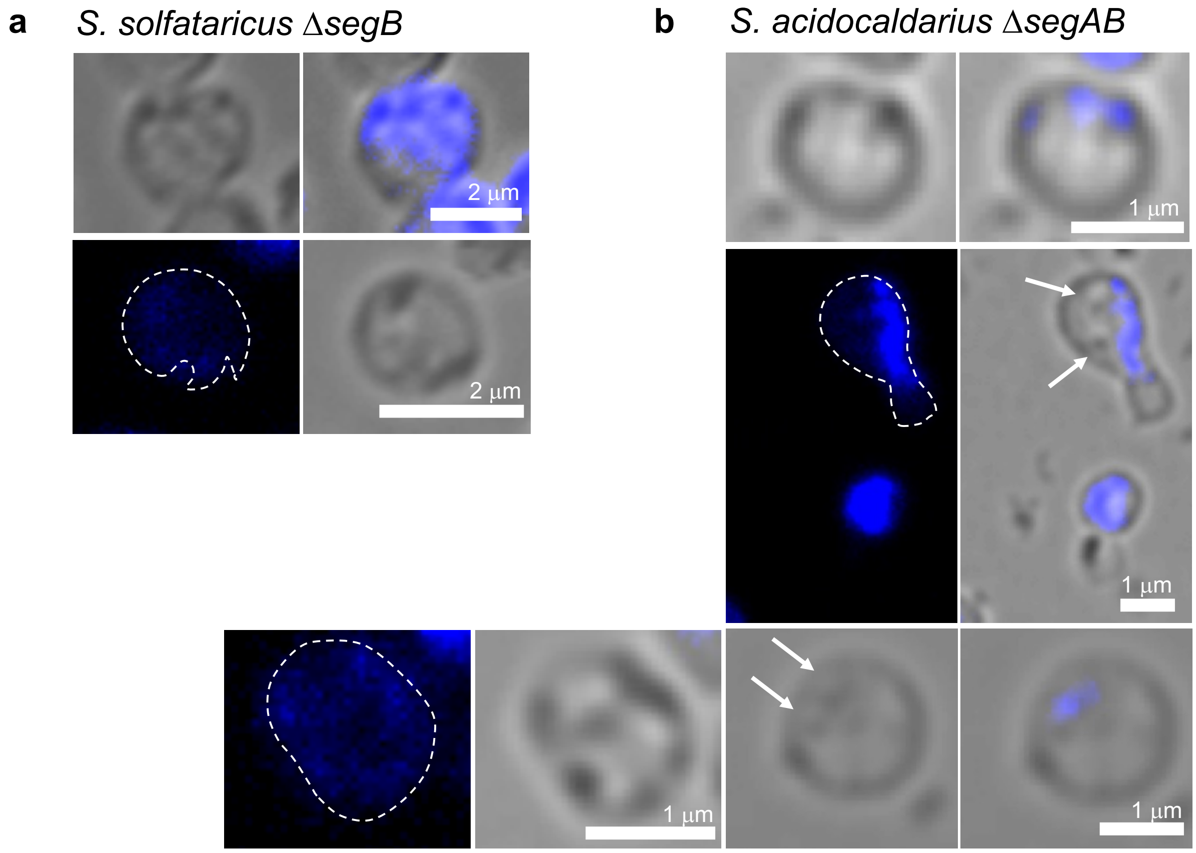

Supplementary Fig. 8. Deletion strains exhibit different aberrant phenotypes. Microscopy images of DAPI-stained cells of *S. solfataricus* (**a**) and *S. acidocaldarius* (**b**) deletion strains. The white arrows point to blebs. The scale bar is either 1 or 2 µm.
At least three biological repeats were performed with similar outcomes.

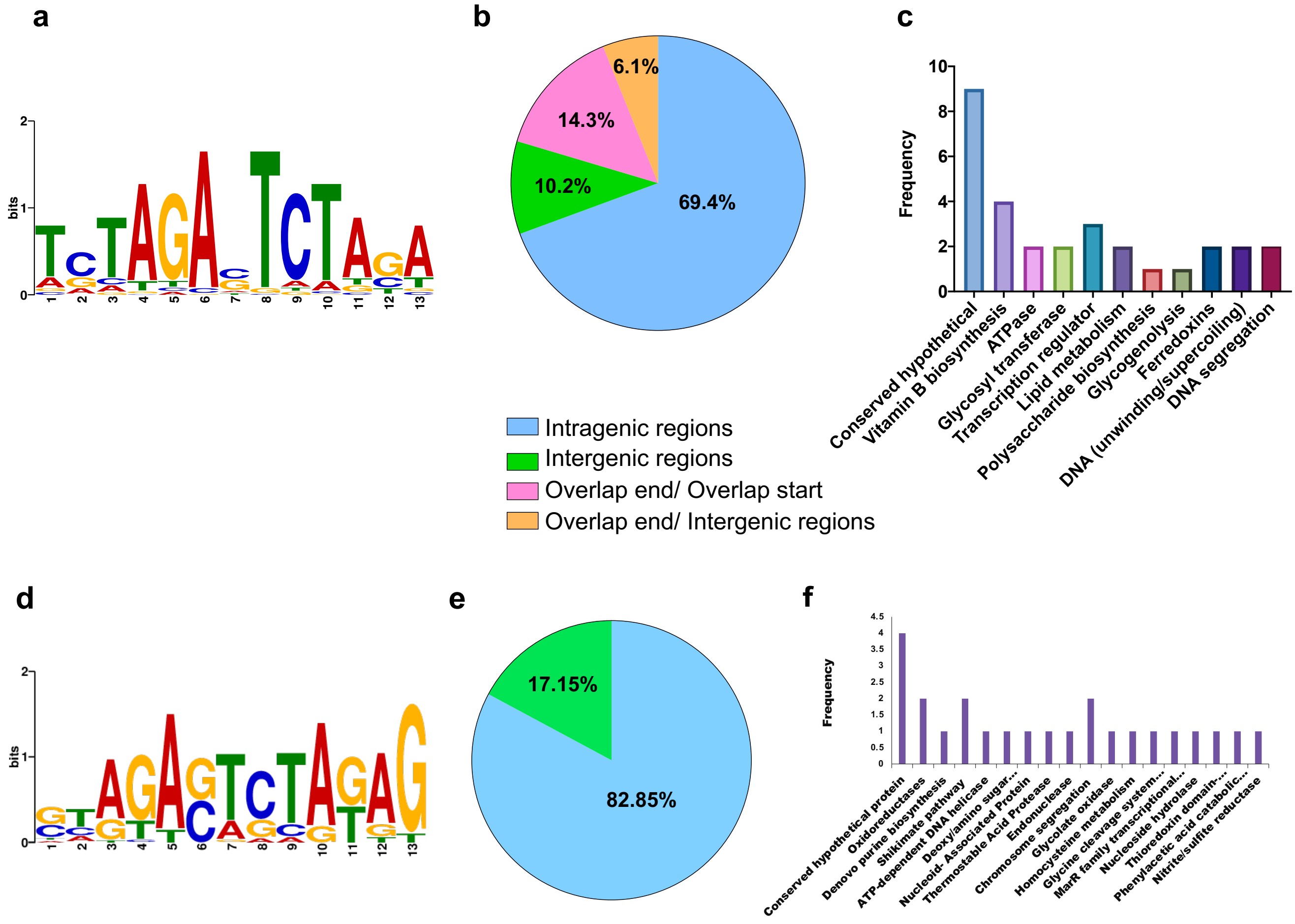

Supplementary Fig. 9. SegB binds to multiple sites on the chromosome of *S. solfataricus* and *S. acidocaldarius*. a DNA-binding consensus motif for *S. solfataricus* SegB identified by MEME-ChIP (with an E-value of 8.3e- 039) based on the 49 sites present in 39 enrichment peaks. **b** Pie chart showing the percentages of *S. solfataricus* SegB sites in intragenic or intergenic regions, or relative to the closest gene. **c** Established or hypothetical function of proteins encoded by genes that are in the intragenic category in panel B. **d** DNA-binding consensus motif for *S. acidocaldarius* SegB identified by MEME-ChIP (with an E-value of 1.7e-019) based on the 35 sites present in 32 enrichment peaks. **e** Pie chart showing the distribution of *S. acidocaldarius* SegB sites in either intragenic or intergenic regions. **f** Established or hypothetical function of proteins encoded by genes that are in the intragenic category in panel E.

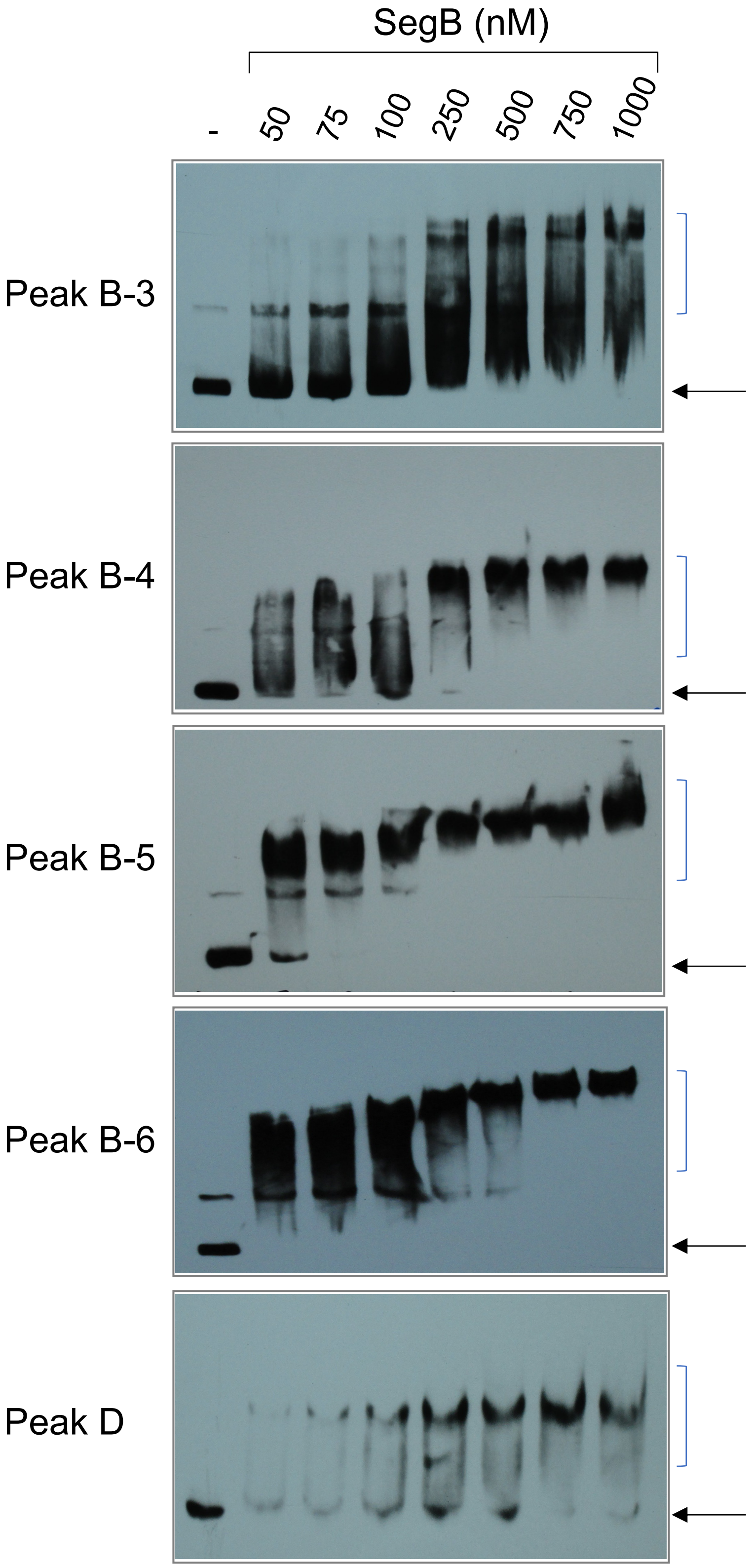

Supplementary Fig. 10. *In vitro* validation of the SegB binding sites identified by ChIP-seq. EMSAs in which biotinylated DNA fragments (1-5 nM) harbouring the sequence corresponding to different enrichment peaks were incubated with *S. solfataricus* SegB in the presence of competitor polydIdC DNA (1 μg). Unbound DNA is indicated by the arrow and SegB-DNA complexes by the bracket. For each DNA fragment two independent experiments were carried out with similar results. Uncropped images are in the Source Data file.

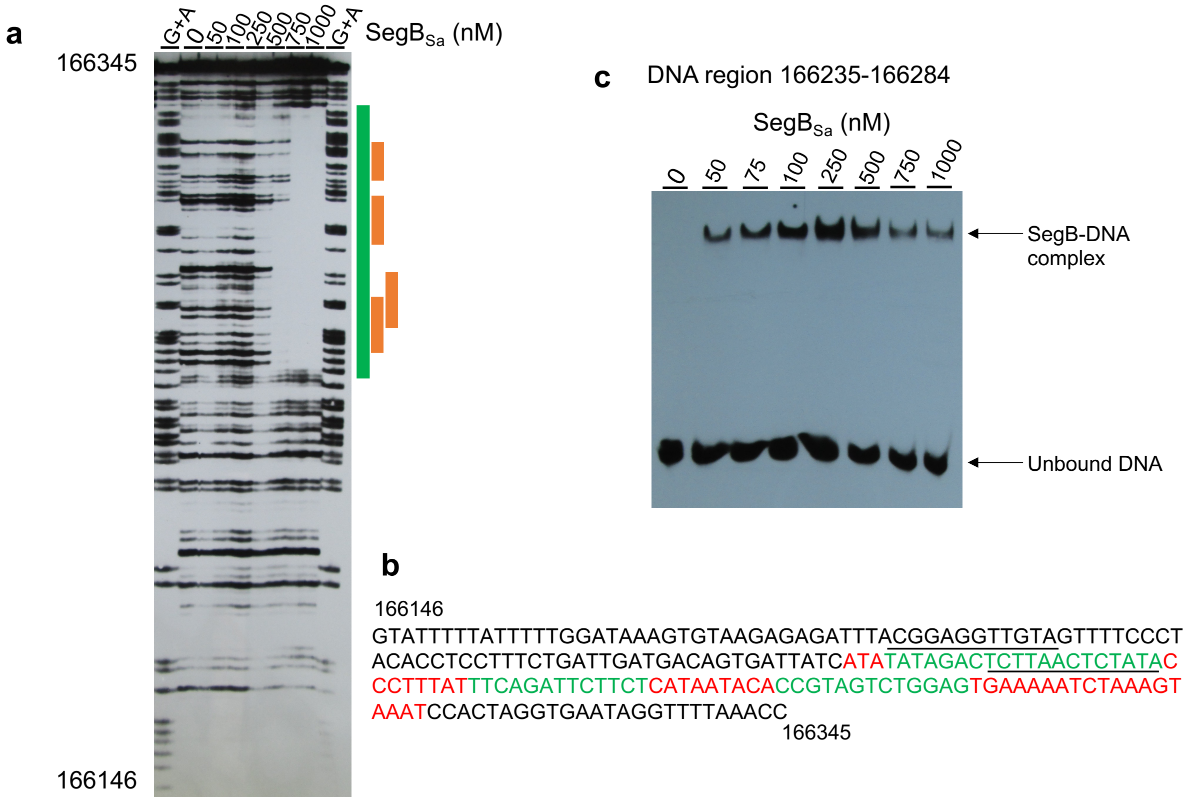

Supplementary Fig. 11. *S. acidocaldarius* SegB binds to the DNA region upstream of the *segAB* locus. a DNase I footprint performed with a biotinylated DNA fragment spanning the region upstream of the *segAB* genes, including part of the *segA* gene, and increasing concentrations of SegB. The window of protection is indicated by the green bar and the potential binding sites are denoted by the orange bars. **b** Sequence of the DNA fragment deployed for the DNase I footprint (genomic coordinates: 166146-166345). The sequence highlighted in red corresponds to the window of protection and the sequences shown in green are the potential SegB binding sites. The sequence closer to the 166146 bp end contains two potential overlapping sites (TATAGACTCTTAA and TCTTAACTCTATA), indicated with a line above and below the text, respectively. **c** EMSA in which increasing SegB concentrations were incubated a biotinylated oligonucleotide spanning the region upstream of *segA*.
Uncropped images of both DNase I fooprint and EMSA autoradiographs are provided in the Source Data file.

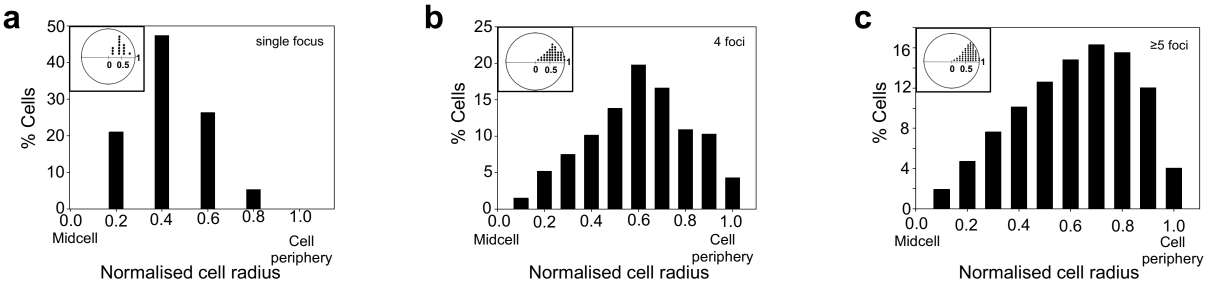

Supplementary Fig. 12. Distribution of SegB foci along the cell radius: foci number increase correlates with a shift of the prevalent position towards the cell membrane. Analysis of the position of SegB foci in cells containing a single focus (**a**) (n=19), four foci (**b)** (n=196) and five or more foci (**c**) (n=557). The inset in the top left corner is a diagram indicating the location of the foci within the cell. Source data are provided as Source Data file.

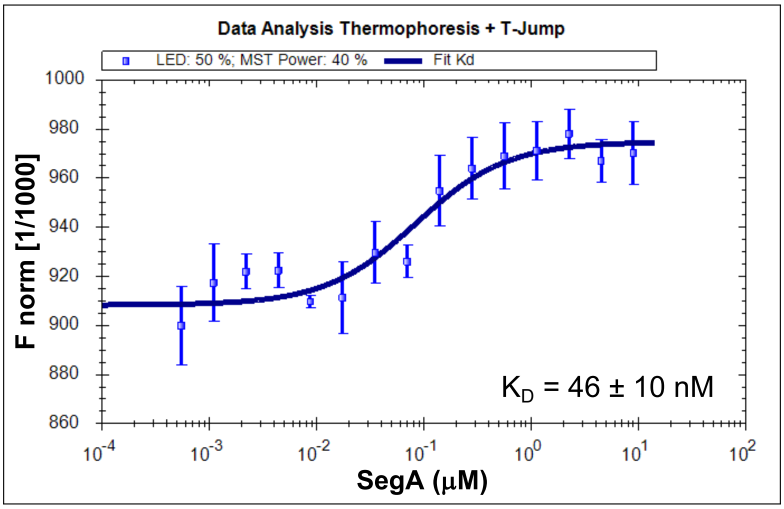

Supplementary Fig. 13. SegA interacts with SegB with high affinity. Microscale thermophoresis (MST) experiments were performed mixing NT-647-labelled SegB (304 nM) with increasing concentrations of SegA (0.6, 1.2, 2.4, 4.9, 9.8, 19.5, 39, 78, 156, 312, 625, 1250, 2500, 5000 and 10000 nM). measurements were carried out using the following parameters: 20% LED power, 40% MST power, 5 second delay before heating, 30 seconds during MST, 5 second delay after heating, 15 second delay. Binding data were analysed using NTAnalysis software (NanoTemper Technologies). The average values from three replicates were used to plot the binding curve and derive error. Source data are provided as Source Data file.

Supplementary Tables

Supplementary Table 1. SegB binding sites identified by ChIP-seq and DNase I footprint on *S. solfataricus* chromosome. ChIP-seq enrichment peak sequences and relative genomic coordinates, with high-enrichment peaks labelled from B to E and low-enrichment peaks designated by Roman numerals (i to xx). Sites determined within the peak sequence through MEME are underlined and highlighted in yellow, whereas those further identified by DNase I footprint are underlined and highlighted in blue.

Peak ID-Fig 3	Start	End	Strand	Position of the peak	Gene accession number/name	Product (gene function)	DNA sequence under peak<br>Motifs identified by MEME (yellow)<br>Further motifs identified by DNaseI footprint (blue)
B-1	1576	1748	+	intragenic	SSO_RS00015 (SSO0002) (*thiD*)	Phosphomethylpyrimidine kinase (Thiamine biosynthesis)	AAATTCGCTACAATGACA<br>GTCAAATACGGTTTGGAC<br>TTAGGAGGAGGATATGGA<br>CCAGTAGATCCCTTTGCC<br>CCTATAGAGTCCATAGTG<br>AAGAGAGAAGAAGGAAGA<br>AATCAGCTAGAAAACTTA<br>CTTTGGTACTTAGAGTCT<br>AATCTTAACGTTATACTT<br>AAACTAATTAA
B-2	14998	15178	+	intragenic	SSO_RS00080 (SSO0016) (*queC* or *exsB like*)	ExsB transcription regulator (Queuosine biosynthesis)	ACAATATATCTGTCTCCA<br>ATAAAAGGTTGAATGTCG<br>TCCTCAGTTTTCTGTCTA<br>ACGTATTCAGTAGTGGGT<br>TCTGCCCTATTATTAGCT<br>ATGACAACCCTAGAGTCT<br>TCATCTAAAATGCCGTAT<br>AGTAATTCCTCTTTTTCT<br>GAGGGCTTTCCTATGGAT<br>TTTCTTACTTTTACTGTA<br>C
B-3	18528	18700	+	intragenic	SSO_RS00095 (SSO0018)	Ferredoxins (Oxidoreductase activity)	AATCTCAGCTGTCGGTAA<br>TACGATTTAGAAAGGATG<br>AAATCGCTTATTCAATTT<br>ACTCTAATAAGCGACTAG<br>AGTCTATAAGAAACACTC<br>TAGAGTATTCTACCATAG<br>AGGGAGAAGGCTTATTCA<br>ATGGATGCATTCTATGCG<br>GTAAATGTGTTAGTGTTT<br>GCCCTTACGGT
B-4	20475	20647	+	intragenic	SSO_RS00105 (SSO0020)	Archaea conserved hypothetical-Predicted nucleotidyltransferase	TTTAAATGTATATCCTCG<br>CCCTTCAAAAATGCCTTC<br>TAAAATCCTTTTACTTTT<br>TAAAATCTTAAATGAAGA<br>ATAGTCTAGAGTCTCTAT<br>TTCAGACTCTTCGACAGT<br>GGATAACGGTTTAGTGAT<br>CCCCTTTTTTCTTAACTC<br>CTCTAAGACGTAATAATG<br>TTCTGGTTTGA
B-5	22703	22875	+	intragenic	SSO_RS00120 (SSO0023)	Archaea conserved hypothetical	GATTTTTATGAATTAGGA<br>TTCACAAATATTAACCAC<br>TCCAGTCTACAATTAATA<br>TTATCTGCTTACTTCAAG<br>AGTCTAGGCTATAGAGTA<br>TTCGTGGAGTATGAGAAG<br>AAAGGAAAACTACTTGAT

							CTCTACGTTAGTGATGAAGATATGGGAATAGAAGTAGAATATGGTTA
B-6	24311	24483	+	intragenic	SSO_RS00130 (SSO0025)	Archaea conserved hypothetical	GCTCGTAATTGAGGCCAAAATTCACAAAAATGATTACCTACAGACTCTAGAGTACTCTAAATATTTCAAGTACGGTATGGCAGTATTTCCATTTACTGGAGAGTGTAGAGTACCTAAAGGTTGGATTTGTATATTTAACACCACAAAGGATCAGTCTAGATTCTACTCTCTTC
B-7	26874	27062	+	intragenic	SSO_RS00145 (SSO0029)	Archaea conserved hypothetical (cystathionine-beta-synthase CBS domain)	ATTTCTAAGATGAAAGAAAACAAGATGTGGACTGTACCCGTTATCAAGGATAGGAAATTAATAGGTTTGATCTCTTATAAAGATCTTCTTTCTAGAAGGGTGAGTCTAGAGACTAAAGCGATAAACATTATGAGTCCCAGTGTTACTGTACAAATTGATGAGGATATTAATAGATTAATTGCAAAATTC
B-8	29213	29385	+	intragenic	SSO_RS00155 (SSO0032)	Archaea conserved hypothetical	ATCGTCTTCCTTAATGCCTAAAGTTTTTGACGCATTCTTTACAAATAGGGTTCTATCAAATTTGTAATCTTCGTATATATTCTTGACTTTAGACTCCAGACTCTTCACTACGTTAGAAATTATCTCATTTTCAAAATATGCATGAATAGCAAAAACTAAACTTAACTTGCTTG
B-9	29661	29833	+	intragenic	SSO_RS00160 (SSO0033) (*segC*)	*sso0033 segC*	ATCTTATTAACGTCCCAAATTTTCTCTATGATTTCCATACTTTTTTTCTTAAAATTTTTACTTACGAATATTCTACTCTCTAGACCCTTAGACTCTAGTAAGTCTTCTGGATCATAACCTTTGTTACTAATTCTTAAAATTATTTTACCTCCATCACAATTAATACAAACTCC
B-10	30052	30258	+	overlap end/overlap start	SSO_RS00160 (SSO0033)/ SSO_RS00165 (SSO0034)	*sso0033/segA*	CAGAATAAATTATTGCACTATAGAGCTTCTCAAACATACTTATACTTTTTAAAAATCTAGCATATAAATATTATCATTATAATATGGTGGACTCGTCAGTATTATCATGTAGAATTCCTTATTAAACGTTATAGATAGAAGAGTCTAGACATGATAGTCACAGTAATAAATCAGAAAGGAGGAGTAGGCAAAACAACGACTTCAGTA

B-11	30551	30723	+	intragenic	SSO_RS00165 (SSO0034)	*segA*	CAATGTTGGTTGCTGACAGAATAGTTTCACCGGTAACACCACAACCCTTAGCACTAGAGGCAATAAAGAATCTCGACTCTAGATTAAAGAGTATAGGGAAGAACGCTTATTCTTTTACAAATTTTTCAAAAAAGGTAGTTAAGCTAGATAATCTATCATCAGTAAAATTCACA
B-12	30730	30909	+	overlap end/overlap start	SSO_RS00165 (SSO0034)/ SSO_RS00170 (SSO0035)	*segA/segB*	ACAATACCACCCTCTAGATTATTCATTGAAGCTTCTAGACTAGGAGTTCCAGCGTTAAGATATGAGGAAGTTAGAATAAAGAAACCTAAGCTAGCTAACTATTATCAGCAATTAGCAAAGGTGATTAGTGAATGAGTGAATTAGATTTCCTATTGAAGAAGAAAAGAAAAAGTGAGGACG
B-13	31166	31346	+	overlap end/overlap start	SSO_RS00170 (SSO0035)/ SSO_RS00175 (SSO0036)	*segB/*archaea hypothetical	TGCAAAAGAGAGATTTAAAGAGTAATATCTGGTAATATTTCTAGGAAGTATTACTAATCAGGTTTAAATGCTAGAGATTATATTTATCTGTAACTGTTAAGGTGATTAGAATGGACATATGTGTAAAGGCTAGAAACGATGAAGAAGCTACAAGGGCTCTACAATTAAATTACAATTGTGT
B-14	31693	31865	+	overlap end/intergenic	SSO_RS00175 (SSO0036)/ SSO_RS00180(SSO0037)	Hypothetical protein/ flagellar hook-basal body protein	AATCTCAAAAAATTAGGATTATATAAAAATAGGATTATATAAAAGGGAGATTTTAAGTCTAGAGAGTGTCACTCTTGACTGAAGAAAGACGAGTATAGAGCGAGGAGTTAGCGGACTTTAAAACCGCGTTGAGTTACAAAAATATATGCATAGATAGCGTTTAGGCATTGAGG
B-15	34685	34857	+	overlap end/overlap start	SSO_RS00185 (SSO0038)/ SSO_RS00190 (SSO0039)	Conserved PadR family DNA-binding transcriptional regulator /conserved Phosphomethyl pyrimidine kinase (Thiamine biosynthesis)	TTAAAGAATGTCTTGTGAAATATATTTGAGTAATTCTAGAGATTTATTTACAACAGTGACTGGATCTCGATCTAGAATGAAGAGTGAAGGCTCTTTACCATAATCACCTAAATCCACAATATAATTAGGAACCTTAGACAACTTACGAATACACGAATCAACCATAAAGTTCA
B-16	43657	43829	+	intragenic	SSO_RS00245 (SSO0049)	Conserved PadR family DNA-binding transcriptional regulator	AAGAGGGTAGAAAAGCTATAGGAGCTATGTCAGAGGAGGATAAAATCAAAGAAGCAATAGAACAATTGGAGTTCTCCGCTAGATACATAGTCGAGAATCTAGAGAAATTAAATGACGAAGATAAGCGAAAAGTGAAAAATATATTGGACGAATTAAGTAAAGTTATGCGATAG

C	120356	120528	+	intragenic	between SSO_RS00685 & SSO_RS00690	CopG family transcriptional regulator/hypothetical (could be socitrate dehydrogenase (IDH))	TTATATGTCACATCTAATATTCCCCCTAGTCTAACAATTTTACTCTTCATTCTGATAAAAATTCAAGTAAGAAGAGTGAAGAGAGAAGAGAGAAGAAAATAGAAGGGAAGAAGAAGAATAAACGCGGAAGAGCGCAAAAGAGAGCGTTTGAACCCACGGGTGGGCCCAATAGC
D	1001960	1002132	+	intergenic	between SSO_RS05720 (SSO1161) & SSO_RS05725 (SSO1162)	Conserved hypothetical containing HEPN domain/MFS transporter (carbohydrate transport)	CTTAAAACAAGAGAAAAGTAGAGAAATATTCTATTATACTCTCTTTTTCCTAAATTTTAATCTTAAACTTAAGGGAAGTAGAGTCTAGACTCACGTTAGGGTATGAAATTATCTCATCCTAATTTTTATTTGGCATAACGCTAATATGAAAAACATAAATAGTTAGAGTGTTA
E	2055588	2055760	+	intragenic	SSO_RS10935 (SSO2241) (*bps2*) (*clsN*)	BPS2 protein homolog - AAA family ATPase (SMC like protein) (ClsN) highly conserved in archaea and in bacteria	TCGTCTATTTGGAACTTAACAGAACCTATCCTATTAATTAACTCTTCCTTTTTAGCCTTTAATTTTGCAATTTCATTCTCTAGAGTCTGTAACTCCTTTAATCTAAGGTCGATTTCTTCTTTCCTTTTCTGTAATCCTATTATATCATTACGCAAACTGTCCAAGAGCGAGGT
i	50316	50488	+	intragenic	SSO_RS00305 (SSO0060) (*gnptA*)	Glycosyl transferase (GlcNAc-1-P transferase)	TACTTGATACTCCTTAAATCGACCCATTTTCATTACTATATGTGTTAATGAATGAGGTTTTTCATCCCAATATAATCTACCGCTAGAGTCTACTTTGCCAAATGATACTCCCTTGAAGTTAGTTCTTAGCTTCAATATAAATTCCACGACGTATGGTATGTAGAGAATTGCTA
ii	109363	109535	+	intragenic	SSO_RS00625 (SSO0129) (*cbiG*)	Protein G (CbiG) (Cobalamin biosynthesis)	TACTATATAATCTATTCTATCGTTTAGAATATAAAGTGATTTTAAGGTCATCCTTAAGGAATAAACAAGAACATCTAGGGGAGTAGATTCTAGATACCTTATACCAATGTAAATTCCAGAAGGTATTAAGTATAGTCTATTACTGCTCTTATCTTCCATACTATCAACATTTT
iii	196087	196259	+	intragenic	SSO_RS01130 (*rpoB2*)	DNA-directed RNA polymerase, subunit B"	ACTATAACTCTTTCAGATCCATTTACTATGAAATACCCTCCTGGGTCTTTAGGATCTTCACCTATCTCAATTAGCTTATCTAGAGTATACTGTGATATTGGGTCTATGGCTGATTTAAGCATTATAGGCAGGTCGCCTATATAAACCTCTTCTGGTTCTGCTTCAATATTATT

iv	235204	235376	+	intragenic	SSO_RS01360 (SSO0274) (*tgtA*)	Queuine/archaeosine-tRNA-ribosyltransferase (Glycosyl transferase)	AGGTAAATCTAATATTACTCCGATATCTGGTTTTATTTTAAGCTGATAATTCACGATCTGGAGATTGGTTATTCCTATTTCTCCATACTCTAGAATTTGATATGCCCCTGAATCTGTCATTATGATCATTTCTTCAGAGCGTAACTCCTTATGGATATCATCCTTTATATAGT
v	254482	254654	+	intragenic	SSO_RS01490 (SSO0298) (*thiF*)	Thiamine biosynthesis related protein	GAAAAGGATACTCATTTAGAACGCATGTCTTACATTGTGAGTTCTTCTCGATGTTAATTCTCTCAATTTTTAATTCTCTAGAATCTATATAGAATAATGAATAATCCGGATTGCCTCTCAAGTGATTAAGCATTAAGTTAACTTGAAGTGTAGCTGTTAATTCTACTATTAGT
vi	717556	717728	+	intragenic	SSO_RS04240 (SSO0873)	Polysaccharide biosynthesis	ATTTTGGGAGGAGTACTCTCAGTCCTCCTTAACTACTCTTTTCTGTCTAGAAAAGTGGGGCTAGTCTTACCTTCTCTAGACTTTGCATTTCTGTTTAAGCACTTCAAGGAAGGCTTACCTCTCTACTTGTCTTCTTCAGCTAATTTCCTCTCATCGCAAGGGGACAGAGTTAC
vii	818763	818935	+	intragenic	SSO_RS04795 (SSO0963) *topR-2*	Reverse gyrase	AAAATTGTTTCCTAACACTACCTTTACTTCATTATCTAATACAAATTTATCTGAAATAAAAATTAAACTGGAAGACTCTAGAAAAGAAGAAGGGAATAGGGTCCATTTTAACATATCAACTGGTTTACTTATAGTAGAGTCTCCAACAAAAGCAAAGACTATAGCTAAAATGT
viii	965294	965466	+	intragenic	SSO_RS05535 (SSO1119)	Highly conserved protein of unknown function DUF1641	TCATAGTCTATCTGATCAATTACTTTCAACACTTTTTCGTCAGTTAGCTTCTTAATTATTGGCGATAATTTCTCTAACGCGGTAAGAGTAGAGTCTATATCAATCTGCGATAGTAATTGCAAAGCCCTTTCGCTTGTTAGTTTTTCTAGTATTGGCCATATTGCTTGTATTTT
ix	1066884	1067056	+	intragenic	SSO_RS06045 (SSO1228) (*tmoA*)	Toluene-4-monooxygenase system protein A (containing YHS domain) - toluene catabolic process (Ferredoxin)	ATATTTGGTAGCGAAAGGATTCTGGCTCTCCGTTAGGCTAATAGGAGCCTTAACCGGAGTTTCAATGGATTACCTCACTCCTCTAGACGCTAGAATAATGTCATACAAGGAGTTTATGACAGAATGGGTAGGTGCGCAGTTAAAGAGATTATTAGAAGATTATGGAATAAGAT

x	1297295	1297467	+	intergenic	between SSO_RS07065 (SSO1444) & SSO_RS07070 (SSO1445)	CRISPR-associated protein Csa3 (Type I-A) /CRISPR-associated transcriptional regulator Csa3	TATAAGAGATTATCTCAGTCTTATTGTCGAGGAGTAGTGGTAGGAGTGATACCTTTCAATTCTATAAGAGATTATCGGAAGAGACAAGACTCTACAGTACAATATGCCCCAGATGCTTTCAATTCTATAAGAGATTATCAACCGGAAGTACGAACTATGTTGCTGAGATTGTG
xi	1699509	1699681	+	intergenic	between SSO_RS09090 (SSO1883) & RS_SSO09095 (SSO01884)	Transposase ISC1234/archaea conserved hypothetical (DNA polymerase beta domain protein region)	GCCAATAAAAATTCAATTTTTTACATTCCGGACACTCTCACGAGTTAGTCCACAATTACAGACTAATAGATATTGAAACTGCTTATATAGATTCTAGATACGGTAATTTAGAATATGAAAGTGAAGACTTGAAATCACTAATTCAAGTTGCAGAGAATGTAATAAAATCACTAGAGG
xii	1910560	1910732	+	intragenic	SSO_RS10195 (SSO2094) (*treX*)	Glycogen debranching enzyme - glycogen catabolic process & protein homotetramerization	TAGGGCACATCCAGCATTCAGAAGGGAAAGATATTTTCAAGGAAAGAAATTATTCGGCATGCCGTTAAAAGATGTGACCTTCTATACTCTAGAAGGTAGGGAAGTTGATGAGAAAACATGGAGTTCCCCGACGCAACTAGTTATTTTCGTGTTGGAGGGAAGTGTTATGGACG
xiii	1999893	2000065	+	intragenic	SSO_RS10630 (SSO2176)	Hypothetical (present in some crenarchaeota only)	AACGGTAAATAACTATATATTGTTGGGGATGGTGATTCATTTTCAGAATTTATCTGGAATATATATATATGTACGTTTTTACTAGTACCGTTTGCTTGATAAAGTATATTGTTGTGATAAATTAGGTTTAATACTATTTCACTCCTATTTAATTTTAAAAATCCCTTATATAC
xiv	2112188	2112360	+	intragenic	SSO_RS11245 (SSO2309)	Cofactor biosynthesis protein (heme biosynthetic process)	GATTGGGCTAGCGTTGTAAACATAACTCGCCATGTTATCTATTATTTTCTCTTTCACCATTTTTCCATTCGACCAACTGAATAGTGAAGGCTCTAGATAACAAGCAAGAGCACATGCATTACAGCTCTCATATTTTTCCCATGGATAACTGTTCCATAATTTTACTATATCGA
xv	2281328	2281500	+	intragenic	SSO_RS12200 (SSO2514)	3-hydroxyacyl-CoA dehydrogenase /enoyl CoA hydratase (Lipid metabolism)	TGGGGTTTTCTTTTTTCTAAGAAAGCTTTCACTCCTTCTTCTACATCTTTAGTTGTGAATAAAAGCCCAAATAATGTTGACTCTAGAGTTTGTCCAGTCCAGATATTAGATTCGTATCCTAACTCTATTGCTAATTTAGCTGCCAATAGTGATATTGGTGATTTTTCAGCTAT

xvi	2377298	2377470	+	Overlap start/overlap end	SSO_RS12665 (SSO2610)/ SSO_RS12670(SSO2611)	Conserved hypothetical/conserved hypothetical in archaea & bacteria	TACTAGTTCTCTCAAAGA<br>TATGGCTTTAAGTTTAGA<br>ATAGAGCGGTGGATAGTA<br>AGCAGCGTAAAAGATAAA<br>AGGTATTCCTTTACCTTT<br>AGAGTCTAGATCCATCAT<br>TAAATATAATTACGTATC<br>AAAAGCATATTAACTTAT<br>CCACAACATAATATTGTA<br>ATTTAACTAAT
xvii	2589275	2589447	+	intragenic	SSO_RS13735 (SSO2828)	Hypothetical conserved in crenarchaeota	AATAATGTGGGTAATATT<br>ATAAGATCCTACATATTC<br>TCTACCTTGGAAAACAAC<br>CGGTAATTTCCCAATATC<br>GCCTTCACATCTATAGAG<br>TCTACTATCAGAGTTTAG<br>GAGAAATTTTGTAACTGG<br>AAGAACTAAAGGGATGAA<br>AAATTCTGGATCGCTTAA<br>TATTTTAATTA
xviii	2630263	2630435	+	intragenic	SSO_RS13955 (SSO2877) (*acd-6*)	Acyl-CoA dehydrogenase (lipid metabolism)	CTTTTTCTTCTGTTCTTC<br>ATTACCGAAAAGCAATAT<br>TGGAGTCATGAATAACCC<br>TCCAACAGATATTCTCGT<br>AGAAAGTGAGGCCCAGAC<br>TCTAGATATCTCTTCTTG<br>AGCTATGGCAGTCATTAG<br>CGTATCTCCACCTTGACC<br>ACCGTATTGTTCTGGTAC<br>TGCAACACCAT
xix	2727724	2727903	+	intragenic	SSO_RS14490 (SSO2984)	Hypothetical conserved in archaea & bacteria (nickel permease)	TTGATATAGGGGCAAACA<br>AGGCTGGGTTATTACAAG<br>GAGCTTTCGTCATAACTC<br>CAACCAAGGTGTTGGTTA<br>TAGTTATCGCTTCCACTG<br>CTTATAGTATACTCTACT<br>CTATAGAGGTAATTTCAG<br>TATTTATAATAGCATCTG<br>CAGTTTCAATTATATCTC<br>TCTCACTATTAAATTTCG
xx	2967544	2967706	+	intragenic	SSO_RS15595 (SSO3218)	ATPase	AAAACCATCGTGATCGAC<br>ATAAACGTTTTTTGCAAT<br>AGATCTCAATGCTTCTGC<br>CATTATGTTGTAATCATA<br>TCTAGAATCTACCCCAGC<br>CAAACCTACATAAGCTAC<br>ATCTGGTTTCATACCTTT<br>TGTGGCTAATAAAATCGC<br>TCTGTTAACGTTTTTTAC<br>A

Supplementary Table 2. SegB binding sites identified by ChIP-seq on *S. acidocaldarius* chromosome. ChIP-seq enrichment peak sequences and relative genomic coordinates. Sites determined within the peak sequence through MEME are underlined and highlighted in yellow and those identified by DNase I footprint are shown in blue.

Peak ID-Fig 5	Start	End	Strand	Position of the peak	Gene accession number/name	Product (gene function)	DNA sequence under peak<br>Motifs identified by MEME (yellow)<br>DNaseI footprint (blue)
1	62063	62362	+	intragenic	SACI_RS00365 (Saci_0079)	Aminoimidazole ribonucleotide synthase related protein	CGTTACAAATGGCGGTAT<br>AAGGGCTACTGCAAATGA<br>GTTGCCTGAAAACTTATC<br>ATTAGCCATAAACACAGA<br>ATCTTTTCTGAGGCTGAT<br>AAACAGAAAAGTGTTAAA<br>TATGCTTAATGAACTGCA<br>GATAGATGTTTTCGGGCT<br>ATCTATAGACTCTATACT<br>CATCTTCACTGATATAGG<br>TAATGAAGTTGTAAGTAA<br>ATTAAAAAGCAAAGGTAT<br>TAATGCGGAGATAATTGG<br>AAAAGTAGTTCAAAGACA<br>AGATTCGCCATTAATTAC<br>TAGTGAAGGTAAACCTCT<br>AATCATGAATTT
2	153097	153396	+	intragenic	SACI_RS00905 (Saci_0188) (*aroA*)	3-phosphoshikimate 1-carboxyvinyltransferase	AGAGGAGATGTTCCAGGA<br>GACTATGCATTAGCTTCA<br>TTTTATGCTATTGCCTCT<br>GCAATAACTGGAGGAGAG<br>ATTCAAATTAAGGGACTC<br>TATTCTCCTCCATATTAC<br>GATGGCGATCACAGCATT<br>GTAAAAATAATTAAGAAT<br>ATGGGTGTAGACTCTAGA<br>GTCGAGGGTAACTCATGG<br>ATTGTTTCAGGGAACGGA<br>ATTATAAAGGGAATAAAA<br>GTGGATGTGGACGATATG<br>CCTGACCTTGCACCATCT<br>ATAGCTTGTATTGCACCC<br>TTTGCTACCTCAGAGACT<br>GAAATTACTGGA
3	154065	154364	+	intragenic	SACI_RS00910 (Saci_0189) (*aroD*) SACI_RS(Saci_0190)	Type I 3-dehydroquinate dehydratase	AAGCATATCTTAGAAAAA<br>TAGCTGTAATAGCTAAGA<br>GAGGTTATAGGGAACTTT<br>TGATGAGAGTTCTTGATA<br>ATTACGATAATGCAGTTG<br>TTATGCCTATGGGTGTTA<br>ATGGAGTAGAGAGAATAG<br>CCTTCTCTCTTCTGGGAT<br>CTAAGTTGATATATGCCC<br>ATGCTGGCGAAGAAACTG<br>CTAAAGGGCAGTTACATT<br>ATAAGGATGTGAGAAGAA<br>TCTTAAATCAACTTTCCA<br>CAATTATGTCTTCACCAT<br>CAACCTGAACGCGGTATA<br>GTTTCAATGGTTTCTTGG<br>TTGCTCCTTTGA

4	15618<br>5	15648<br>4	+	intrag<br>enic	SACI_RS009<br>25<br>(Saci_0192)	ATP-<br>dependent<br>DNA helicase	CTGACTATCTTCTAAAAA<br>TATACTTTCAGGCTAAGG<br>CAAATGTTTTGGTAGTAT<br>TTCCGTCATACGAGATAA<br>TGGATAGGGTTATGTCTA<br>GGATTTCATTACCTAAGT<br>ATGTTGAAAGTGAAGACT<br>CTTCAGTAGAGGATCTAT<br>ACTCTGCAATATCTGCAA<br>ATAATAAAGTCCTGATAG<br>GGAGTGTAGGAAAAGGAA<br>AATTAGCCGAAGGCATAG<br>AATTAAGGAACAATGATA<br>GAAGTTTGATTTCTGATG<br>TGGTAATTGTGGGTATAC<br>CTTACCCACCACCTGATG<br>ATTACTTGAAAA
5	15849<br>3	15879<br>2	+	intrag<br>enic	SACI_RS009<br>45<br>(Saci_0196)<br>(*mpg1*)	NDP-sugar<br>synthase	TTCCTAAATTATATAATG<br>GTATCTGCTATAGTTCTT<br>GCAGGTGGATACGCAACT<br>AGACTAAGACCATTAAGC<br>TTAACAAAACCTAAAGCA<br>CTTCTTCCTGTACTAGGA<br>AAACCATTAATGGACTAT<br>ACACTCTACTCTCTAGCA<br>TCTTCAGACGTTGATACG<br>ATATATCTCTCCTTGAGG<br>GTCATGGCTGATAAAGTT<br>CTCGACCATGTTAAGCAG<br>TTAAACTTACAGAAAAAC<br>ATAGTTTCCGTTATAGAG<br>GAGAGTAGGCTAGGAGAT<br>GCTGGTCCACTCAAATTC<br>ATAAATTCAAAG
6	15960<br>3	15990<br>2	+	interg<br>enic	SACI_RS009<br>45<br>(Saci_0196)<br>(*mpg1*) /<br>SACI_RS009<br>50<br>(Saci_0197)	NDP-sugar<br>synthase/<br>conserved<br>hypothetical<br>protein	TTAATCTCAGTAGAATTT<br>GTCAATGAAATTGCCAGC<br>ATTACTCCCTATTTCTCT<br>TTTTATAACTATCCTAAT<br>CCCACTCTGCATTTTTTC<br>GATTGTCACAGAGTCTTC<br>AGTACATACTCTAGAGAT<br>CCTAATAAACGGTCCCTT<br>ACAGGTAACTATAACGCA<br>GGGAAATAATTTCACTGT<br>AATTAGCAAAACTACCTC<br>ATTGAGATTACAGGGTAA<br>TATAACTATTCAAGCATC<br>AACCCTTCAACCTGGATA<br>CCAGATATTCATTAATGG<br>CATCAAGGCAAATACGCT<br>AACCCTCAATTC
7	16070<br>0	16099<br>9	+	intrag<br>enic	SACI_RS009<br>55<br>(Saci_0198)	Thioredoxin<br>domain-<br>containing<br>protein	TAGGGTTGGTTTCAAGAG<br>ACTTCTTAATGAGATTAT<br>AAGATTATGGAAGAATGA<br>GAGGGATAAAATATTTCA<br>GACTGCTAATTCTCTTCA<br>CTCTTCACTTCAGAATCT<br>AGGATATCAAAAAGTTGA<br>AGCCCAATGGGATCAAGT<br>GGAGAGCATAGTGAGTTA<br>TATTGCATCAAATTTTGA<br>CTTTCAAAACGGTGGACT

							CTTAGGTAGTATGAAATT TCCTCACCCAACTATAGA TCAGCTCTTAATTGCTTA CTCTTTCTACACAAAGAG TGATACTGAGGCTAAACT TTCTATGTTTAC
8	161351	161650	+	intragenic	SACI_RS00955 (Saci_0198)	Thioredoxin domain-containing protein	CAATGTTGATACTGGAAA AGAAGTTGAAGGCAGAAA AGTGTTAAGAAGGAATTA TGATTTAAGAGAATTAAG TAAAAGGTTCAAGGATCC AATAGGTAAACTGAATGA TGTTAGGGAAAGATTAAG AGTCTATAGAGAAGAGAG AAGAAAGTATCCCTTTAT CGACACAAATGTATATAC TCACTCTAACTGTAGAAC AGCTGAGGCTTTAACATT AGCTTATCCAATTACAGG TAAAGGTTTAAATGAAGC CCTTAAGGTAATAGATAT GATAAACACTAAGATTAC TAGAAGACTAAC
9	161892	162191	+	intragenic	SACI_RS00955 (Saci_0198)	Thioredoxin domain-containing protein	TATTCAATAGATTCCCCT TACATTGCAGGTGTAACA TTTAACTCAATGGCTTTT GAGAAAGGTTTAGCTCAT ATTGTGGTAGTTGACGAA AAAGACGGTAAGGCTAAA GATCTTCACCTATCTGCA CTGAAAATATATCATCCT TTCAAGGTCGTGGAGCTC GTGAGTGAAGACTCTATA GATATGCTTCCCTCTTTT ATAAAATCCATGGTTAAT TACAATAAAGGTTCCAGT AGAGTTTATGTGTGTATA GGTAATACTTGTAATTTA CCGGTAGACTCTTCAGAG AAGATTAGATTA
10	162780	163079	+	overlap end/ intergenic region /overlap start	SACI_RS00960 (Saci_0199) / SACI_RS00965 (Saci_0200) (*nucS*)	Short-chain dehydrogenase/ SDR family NAD(P)-dependent oxidoreductase/ endonuclease NucS	GAAAATTAGGTGATGCAG GTGCACCGCCTGAAGATT TTGCTCAAGTTATAACAT GGTTATTGAGTAATGAGG CACAATGGGTTAATGGAG TAGTTATTCCAGTTGATG GCGGTGCTAGACTGAAGT GGGTGTAGAGTACATTCT TCTCTCTAGAGTGAATAT ATATTAGTTTTTTATAAG TAATACTATGTTCAAGGT ATTACTCGAGCCTGATTT ACAGGAGGCTTTAATTTT CCTCAATGAATCGGTGAA TGCTCTACTTACAATTTA TTCTGAATGCGAAATCTT ATATTCAGGCAG

11	163122	163421	+	intragenic	SACI_RS00965 (Saci_0200) (*nucS*)	Endonuclease NucS	CAAACCAGATGGGAGTGTTATAATCCACGGACCTACTAAAAGAGAGCCTGTAAATTGGCAACCTCCAGGATCGAGAATAGAGTACAGTATAGAGAGTGGAGTATTGACAGTAAATGCTGAAAGGAAGAGACCAAAGGAAAGACTCTCCATTCTGCACCACAGAGTCTACTATATTACCTCTTCAGAGGTTAAGCCTGGAGAGTTCTTTCTAGTGGGAAGAGAGAAGGATGAAGTGGACTTCATTATAAATAACCCTGATGTAATAGAGGGTGGATTTAAGCCAATTCACCGAGAGTATCG
12	165363	165662	-	overlap end/overlap start	SACI_RS00980 (Saci_0203) / SACI_RS00985 (Saci_0204)	*segA/segB*	TGAGTTTGGACTACTCTTCACTTCCTCTAAATCTTCCCTAGGGCTTTTTGAAGCGTCATTCTCATTAACCTTTTCCTGGGTTTCACTAGTTTTGGTCTCTGCTAATTTCTGGGTAGTAGACTTGGAAGTCTCCTGTCTGGACTCAACTTTCTGTTCACTAGCCTCTACTTTCTGTTCAGTCTTTTTTCTGTTTAAGATTAAGTCTAACTCACTCATCCTTCTATCACCACTTTAGCTAAGTCTTCATATAATTGTGAAAACTTAGGTCTCTTCACTCTAAATTCTTCATATCTAAGGGCT
13	165663	165962	-	intragenic	SACI_RS00985 (Saci_0204)	*segA*	GGAACTCCGAGTCTAGTGGCTTCAGAGAAAAGTTTAGACTGTGGGATTGATAAGTCTATACTCTTGACTGAAGGTAGCTCAAGCTTTACAGATTTCTTACTCATGTTCGTAAATGCTATTGCAGGTTTCCTTAGACCCTGCAGTCTAGAATCCAGATTCTTGGCAGCCTCTAGCACAAGGGGTTGTGGGGTAATTGGTGTAACTATCTTATCCCCAGCAATCATGGCAGAGACTGCTAATGTGCCCAAGTTAGGGGGAGTATCTATGACTAGAAAGTCAAAGTTCTCGGCAAGTTTCTTA
14	165961	166160	+	intragenic		*segA*	TAAGAGAGGAGACTATAGACTCTATATCACCATTGAGCTCTAGTTTGAGTAGACCAATATGTGCAAGAAATACTTCCACGTTAAATATATTCACACTTTTTCCTCCTAATGGATATTCCCTTTTTTCCCTCTTTATTCCAAATGATATTGTAGCTCCACCTTCGGGATCAAGGTCTAGTA

							ATGCCGTATTTTTATTTTTG
15	166138	166437	-	overlap end/ intergenic region /overlap start	SACI_RS00985 (Saci_0204) / SACI_RS00990 (Saci_0205)	*segB*/(Fe-S)-binding protein (oxidoreductase)	GTAATGCCGTATTTTTATTTTTGGATAAAGTGTAAGAGAGATTTACGGAGGTTGTAGTTTTCCCTACACCTCCTTTCTGATTGATGACAGTGATTATCATATATAGACTCTTAACTCTATACCCTTTATTTCAGATTCTTCTCATAATACACCGTAGTCTGGAGTGAAAAATCTAAAGTAAATCCACTAGGTGAATAGGTTTTAAACCCATTTTTATAAAGGCGAGTGTGCATGACGGATTTGTTGAAATCACAGTATTAACCCCACTCTTCATTACTTTCTCCTTCTTTCTACTCATTA
16	166775	167074	-	intragenic	SACI_RS00990 (Saci_0205)	(Fe-S)-binding protein (oxidoreductase)	GTTCTCACCTACATGTGAGTGAGCTAAACCACAGCAAGTATTAATGACTTTTACGTTGTACCCTAGACCTTTGAAGTATTTCACTGCTTTCTCTACACTACTTCTAGATATAACTGAGGTTAAACATCCTGGAAATATTATTAAATCTTCATTCTGGTCTCTGTACTCTAGAGGAAGGCTGGTCTCCACTCCCATTCCTATTTTCAGAGGTAAGGCTAATGATGAGGGTTTTTCAAGTAATCTTAACATAGATCTTTCCAATGGGTTAGACTTTCTTGAATTTACGAATATCTCTGAGTA
17	167624	167923	-	intragenic	SACI_RS00995 (Saci_0206)	FAD-linked oxidase C-terminal domain-containing protein (glycolate oxidase)	ATCTTCGCTAAAACTTCAGGTAAGTTACTTCTCAACACATTACAGTCTAAGGTGATATATGCCGGTGAGATAACTCCCATAGCCGGAAAAGCTCCCCTTCTTGCACTCCAGAACTTTTCTGGTTCCTTTGGATTAATTATCTCACCGTTATTTTCGCTTATTACATCCCTGACTCTACTCTCTTCACTCTTCACTTGAATCTCTGAACCATCTAGTTCTATAAGCAATATCGCCTCAACTTCAGGTAAGCCTGCCCTATATCTACTCTTTTCTATTGCAATTATGGAATATCTGTCCATC

18	168425	168722	-	intragenic	SACI_RS00995 (Saci_0206)	FAD-linked oxidase C-terminal domain-containing protein (glycolate oxidase)	CCGCTGAGACTAGTTCCAGAACCTCTAATTATAATTTTTCTTCTATTCTGTATCAAGTATCTTATTACAGCAATTGCCTCCTCTTCATTACCGGGTAGTACAACTAATTCTGGTTCTCCCTTTACTGCTGTTAGACCATCAAAACCATAGAGTCTTCTCTCTTCATCCTTTATGACCCACTTGTCCCCAACTATTTGTTTTAGGTCTTGGATCAACACTACAGTTTCACTTTATGATTTAATGGAAATAAATTTTTGTTAACAATTAATTAGTAGGATTAAATTATAGAAAGAACTTT
19	169202	169501	+	intragenic	SACI_RS01000 (Saci_0207)	FAD-dependent oxidoreductase	ACCAAAATTCTCCAGTGGCTATAAGATTTGGAAATCACTGTCAGGATCACTAGGATTATTAGGAGCCTATTTAGAGATCATTGTAAGACTGATACCTAAACCAGAGAAGATAATGTATGCTGAAATAAACGACGTAGATAAGGTATTAAATGAGAGACCGTGGGGAATACTCTTCTCTGCAGACTCTGGAGAAATAAAGAAATACGCTATATTCGCTGGGTTTCAAGATTACTTGAGGAGTGTAGAGAAGGAGTACAATATTTCTCTTGTTGATGGCATTCCTAGCCATGATCTACAGTG
20	174483	174782	-	intragenic	SACI_RS01025 (Saci_0212)	Cystathionine beta-synthase domain-containing protein	ATGGAGTCTATTATTGCCCTTGAATTGCCCTCCTTAAGTTCACCAGTTAACTTAGCCTCTATCGGCATAGAAAGTTCGAATTTCCTTGCAATAACTTTAAGCGCATCAATTCCGCTAAACATTCCCACAATCATGTTACCTTCTAGTACTGGCATACCTGATATCTTTCTGGTGAGTAGAGTATTCACTGCAGAGTGAAGATTCTCTGCTCCATTTACAGTTATTACGGGGTAGTTCATTATTTCCTTTATCGGCATTGCCATTAATCTCTCTTCCTCAGTAAGTATGGAGGACTTCTTC
21	296995	297294	+	intragenic	SACI_RS01710 (Saci_0350) (*gcs2*)	Glycine cleavage system protein GcvH	GGTGAAAATAGAGGGAAGTGATATTGCGGTAGTTGGTATAACGGATTTAGCACAAACCATGGCAGGGAAAATTGTGAAGATAAGGATAAAGAAGAAAGGTATAAAAGTAGAGAGGGGAAGACCTGTAGCAACTCTAGAGAGCGGGAAATGGGCTGGACCAGTGCCTGCACCAGTTTCCGGTGAGGTAGTGGATTCCAA

							CTCAGAAGTTGAAAAAAGCCCTGTGATCTTAAACAGAGATCCTTATGGTCAAGGATGGATCGCAAAGATTAAAATTAGTAACCAGGAGGAAGTTAAGCAATT
22	404080	404379	+/-	overlap end/overlap start	SACI_RS02330 (Saci_0482) / SACI_RS02335 (Saci_0483)	MarR family winged helix-turn-helix transcriptional regulator/ helix-turn-helix domain-containing protein	TGGGCCTGATAGAGGTCCAGGAAGAGAAGACGAGGGTAAAGAGCGTAAAGAAAATAGCCAAACTAACAGACAAGGGCCGCATGGTCTTTGAGAAGATTCTAGAGTTAAACCAGCTCCTCTCAGAAGACAAAGCATGAATAGGCGTCTTACGAGCTCGATAAAGACTTCGAGGCTTTTTTCATTTCGACGATCCTTTCAATCTCAACTAATAATTGAGCGACCTCTTTCCCGCTCTCTGTCAATATTACGAACCTGCGGCGTGGGAACGAGTGCTCTCTTTCTTCATTAACGAGGCCTAAC
23	502140	502439	+	intragenic	SACI_RS03005 (Saci_0632)	Conserved hypothetical protein	GAACATTATTTAGCTGAGGTCTACTTCTCGTTCTGTCCACACTTTGAGCCTAACCACAGGGCGGAAAGTGTAGTAGAATGCCAGTGGCTCTTCTATGATATCGATAACGTGAACGAAACGGTGCTCAGGAACCTCTCTAGACTCTCGCCTAGACCAGTTGTGATCCTCTATTCGGGGCATGGTGCACACTTATATTACAAGTTGTCCCGGAAAGTACGCACAGAAGAATACAAGAAGATCTGGCAAAGATTCGCTAAGCGTTACTTGACACAATATCTAAATAAGATAGATAAAACGAAA
24	580288	580587	+	intragenic	SACI_RS03450 (Saci_0724)	Membrane protein	GAACATAGCTAGGTTGAGCCTCTCAACGCAGGAAGACTCCGAAGCGCAAGAGGAGGAGCTAAAGTGCACAGCCAACGGGATCTCAAGCACCGCGTATAGTGCGGAAAACCAGAGGTTTGCCTACTACCCAGCAATAGCCCTTCCATTTAGGGCAACTAGGGCCTCCGCCACGCAGTGCGGGCCCTTCTACTGTCTAGACGTCGACCCTAACAAGGCCTTCGGCCAAGGGATCCTAGTGAGAATAAACTACGAGGGGAGGAACATTTACTTCTACATAGGCTGGGTACCAGCAGTTAAGGG

25	580496	580795	+	intragenic	SACI_RS03450 (Saci_0724)	Membrane protein	AACAAGGCCTTCGGCCAAGGGATCCTAGTGAGAATAAACTACGAGGGGAGGAACATTTACTTCTACATAGGCTGGGTACCAGCAGTTAAGGGATCGACTATCTTGGGAGTAGTCCAGGGAGGTAAGTCCTTCAAGTCCTTATCCTTGGCAAAGTACTTAGAGGAGTTCGGCATGGATCTGAACACCGTGGGGGGAAAGCTCGGAGTAATCCCTGTTTACCCGCTGTCTAAGGACGTGGATGTGTGGTACAACCCGCCAATTTACCAAGGCAACACAACTATCGCCTCAATAATACCTTGG
26	1007000	1007299	-	intragenic	SACI_RS05660 (Saci_1187) (*iunH*)	Nucleoside hydrolase	GCAATATCAACTATGGCGTCTAATGCCTTTTTGTTCTCAGGCTTGAGTTTTTGTGGCTTTGCAATTTTATCCCCTATTCCTCCTTTACCATGAACCTCTTCTACACTCCTAAAGTCCTTGACCAAAGGCTTATCACTGCCAGGATAGACCTTAACATTTTCCTCTCCTATATACTCTAGAGCCCATAACGCATTATTAACCTCTTGTTGGTAAGAAATGTTCCCCTCAACAATTGTTACCCCTTCCACACTCACTTTGTGTCTCAAAAGCATGAACAAACTCATTATATCGTCCTCTGCG
27	1130248	1130547	+	intragenic/intergenic	SACI_RS06315 (Saci_1322) (*alba*)	Alba Nucleoid-associated protein (NAP)	TAGGTGGTTTAAGTGAGTTCAGGCGCAACCCCAAGCAATGTAGTATTGGTAGGTAAAAAACCTGTAATGAACTATGTATTAGCAGCCCTAACTCTACTAAATCAGGGTGTAAGTGAGATAACAATAAAAGCTAGAGGTAGAGCAATAAGCAAAGCAGTTGACACTGTAGAAATAGTGAGGAACAGATTCTTACCAGACAAAATAGAGGTAAAAGAGATTAGAATAGGAAGCCAAGTTGTAACCAGCCAGGACGGAAGGCAGTCTAGAGTTTCCACAATAGAAATAGGAATAAGAAAAAAG
28	1309139	1309438	+	intragenic/intergenic	SACI_RS07310 (Saci_1534)	Thermostable acid protease	TATAACGCCTCTCCTGGAGAGCAGGTAACAATTAACGGCCCGATATACGAGAAACTCGTAGTCCAGCTCAACTACACGTTCCTTGAGGAGTTGGTTATACCCATTGTAACGGCTGTCGTGATTGTTGTAGCGATAACGAGGAGGAGATAGACAGTGAGTTAATGAATAGAAGTTAGTACGCTAAACCCTTTTTCCTCG

							TTCTAGAGTCTGCATAAT ACGAGAATACAAAGGCAA TAAACTGGAGATTTCAGA GGTGCCCTTGGTCTCAAA CGTTTATAACTTCTTGTT TAACCCATCACT
29	1429094	1429393	+	intergenic/ intragenic	SACI_RS07975 (Saci_1671)	Conserved hypothetical membrane protein	GTAAATTTTTAAATCATT TGGACTAAATATAATATA GAGAGAGTGAAATGACCA AGGTTGAGGTGATTAAAA TTGAGATCAGTGAGCCAT GCGGTCGTAAGCTGGACT CTCTGGAGTCTAGCGCTA TATCTCACACTTCCCTAT CTTTCTTCTGCCATCGAA CACTCAATTAATAATCCA CTTTTTGTGGGTCCCTTT TACATAATCTCCTCAGGT ATTGGTAGTCTAAGTTTA TTAGCTCTTGAACGTCAC GTGAAACTGCTATCAACT ATAGGGTTAGCACTTTCA GGCATAGGACTG
30	1486672	1486971	-	intragenic/ intergenic	SACI_RS08250 (Saci_1727)	DUF1028 domain-containing protein	ACCTAATTGTCTTTTTTC TTTAAGCTGGTCTGCAGA AGTTAAACTATTCACCGT ACTCCTTGCGTCATAACC CTTCTCTAGGAGTTCTAA TCCTTTTTTACCATACTC TAGATTAGCTAGGGCTTG AGTTGCTATTGCCCCTAC CTCTGGTTTCAACCAAGG GACAAAAGCACCCACTGC TAAGAACTTACTGGCAAC ACCTACTCCCCATGCTTC CTCGTTTGGGTCGTAAAT TACTATTGAGTATGTCAT ATGTGTAATATATACTAC TGAAGGTATTTTATAATT ATATAATTCTCT
31	1531730	1532029	-	intragenic	SACI_RS08465 (Saci_1770)	Phenylacetic acid catabolic protein	TCATCAGAATTACGTTGT AGCTGTCAATTACGAAAA CAAACGCCACGGCGTCCT CCCATGTAAATAAGGGCA AATTGAAGGGCTCTAACC TATGTAATCCAAGCCTCA GTTCTTGTAGCTCTTTAA CCTTATCCTTTTCACCAA AGTCCTCGATCATACGAG ACATCTGCCATGCGTGAT TCAGCTCATCTGCGACAA ACCTAGCAGTGAATAGTC TGGAGTCCACAGTTGGGG AGTTAACTAGCCATGGAG ATGTTTGCTCCACAATAG CTAGTTTAGAATCGGCGA TCACAAAAATTA

32	2039264	2039563	-	intragenic	SACI_RS10640 (Saci_2201) (*sir*)	Nitrite/sulfite reductase	CCCAAGTGTAAATCCCTTATTAGATTCACCTTAACTACTGGACCTAATTCCACACCACTATATTCCTTTATTTTCTCTCTCATCCAGATAATCCCAAATTTATGAACAACGTATTTCAACCTAGCCCAATGCCTGTTCTTCCTATCCCCCCAATCTTGTTGTATCCTGACTATAGAGTCTAAAACTTTCAATAGGTCGTCCTCACTTACTGTACCCAAAGGAAGTGCTAAAGCAGAAAATGTCGGATAACCATTATTCTCGCCCATTCCTCCACCAACATACAACTGATATTTCTCTATC

Supplementary Table 3. Oligonucleotides used in this study.

Primer name	5'–3' sequence	Details
segBKOUSforward Kpn	CCCGGTACCGCATATAAATATTATCATTATAATATGGTGGACTCG	Forward primer to clone the 823 bp region upstream of *S. solfataricus segB* into pET2268 for knock out construction
segBKOUSreverse Nco	CCCCCATGGTTTCTTTTCTTCTTCAAT	Reverse primer to clone the 823 bp region upstream of *S. solfataricus segB* into pET2268 for knock out construction
segBKODSforward Bam	CCCGGATCCCCCTACACTATTTACCATTGCAGAAAAAATTGC	Forward primer to clone the 1150 bp region downstream of *S. solfataricus segB* into pET2268 for knock out construction
segBKODSreverse Not	CCCGCGGCCGCCCCTTTTTCATGTTAAAAATTCACG	Reverse primer to clone the 1150 bp region downstream of *S. solfataricus segB* into pET2268 for knock out construction
saci0203 pyrEF for	AGCTAAAGTGGTGATAGAAGGATGAGTGAGTTAGACTTAATCTTAAACAGTTTGAGCAGTTCTAG	Forward primer for PCR to knock out *S. acidocaldarius segB* (*saci_0203*) gene by insertion of *pyrEF* cassette
saci0203 pyrEF rev	CCCTACTTTGGTAATATTCATTATAGTAATATCATTAACTCTTTTTACTCGACCGGCTATTTTTTCAC	Reverse primer for PCR to knock out *S. acidocaldarius segB* and *segA* (*saci_0203* and *saci_0204*) genes by insertion of *pyrEF* cassette
saci0203_saci0204 pyrEF for	GAATCTGAAATAAAGGGTATAGAGTTAAGAGTCTATATATGATAATCACTGTTTGAGCAGTTCTAG	Forward primer for PCR to knock out *S. acidocaldarius segB* and *segA* (*saci_0203* and *saci_0204*) genes by insertion of *pyrEF* cassette
saci0204(-47) FP	BTN-TTATGAGAAGAATCTGAAATAAAGGGTATAGAGTTAAGAGTCTATATATG	Biotinylated forward oligonucleotide containing the sequence 50 bp upstream of *S. acidocaldarius segB* (*saci_0204*) start codon used for EMSA
saci0204(-47) RP	CATATATAGACTCTTAACTCTATACCCTTTATTTCAGATTCTTCTCATAA	Reverse oligonucleotide containing the sequence 50

		bp upstream of *S. acidocaldarius segB* (*saci_0204*) start codon used for EMSA
segA-stop-Rev	CTCGAGTCATTCACTAATCACCTTTGCTAATTGC	Reverse primer containing stop codon for PCR amplification of *segA* and cloning into pET22b (XhoI)
sso35-Forw	GAGGAAACCATATGAGTGAATTAGATTTCCTA	Forward primer to verify the *S. solfataricus* Δ*segB* deletion strain by PCR
sso35-Back	CACACACTCGAGCTCTTTAAATCTCTCTTTTGC	Reverse primer to verify the *S. solfataricus* Δ*segB* deletion strain by PCR
saci0204-seq-for	CAAATCCGTCATGCACACTC	Forward primer to verify the *S. acidocaldarius* Δ*segB* and Δ*segAB* deletion strains by PCR
Saci-Forward	GTAAATTCCATGACGTCTGTGAA	Reverse primer to verify the *S. acidocaldarius* Δ*segB* and Δ*segAB* deletion strain by PCR
AK50	ATGATAGTCACAGTAATAAATCAGAAAG	Forward primer to amplify *segA* from *S. solfataricus* P2 for RT-PCR
AK51	TTACTCTTTAAATCTCTCTTTTGC	Reverse primer to amplify *segB* from *S. solfataricus* P2 for RT-PCR
AK52	TCATTCACTAATCACCTTTGCTAATTG	Reverse primer to amplify *segA* from *S. solfataricus* P2 for RT-PCR
AK53	ATGAGTGAATTAGATTTCCTATTG	Forward primer to amplify *segB* from *S. solfataricus* P2 for RT-PCR
AK80	ATACATATGAGTGAATTAGATTTCC	Forward primer to amplify *segB* from *S. solfataricus* P2 (NdeI) for cloning into pSSR vector
AK81	ATAATAATCGATTTACTCTTTAAATCTCTC	Reverse primer to amplify *segB* from *S. solfataricus* P2 (ClaI) for cloning into pSSR vector

AK87	ACGCTATGTAGGCTGAAGCTA	Forward primer to amplify a 289 bp DNA fragment from *S. solfataricus* P2 for AFM experiments
AK97	AGTAAGATAAATTTACTGAAGTCG	Reverse primer to amplify a 289 bp DNA fragment from *S. solfataricus* P2 for AFM experiments
AK93	ATAATAGCATGCAAGGGAAGTAGAG	Forward primer to amplify a 1028 bp DNA fragment (containing peak D) from *S. solfataricus* P2 (SphI) for cloning into pUC18. The plasmid was used in AFM experiments.
AK94	ATAATAGGATCCAGACTGGCTAAGAC	Reverse primer to amplify a 1028 bp DNA fragment (containing peak D) from *S. solfataricus* P2 (BamHI) for cloning into pUC18. The plasmid was used in AFM experiments.
AK95	ATAATAGGATCCAAGGGAAGTAGAG	Forward primer to amplify a106 bp DNA fragment (peak D) from *S. solfataricus* P2 (BamHI) for cloning into pNER803. The plasmid was used in AFM experiments.
AK96	ATAATAGGTACCTAATTAATAGTTAACAC	Reverse primer to amplify a106 bp DNA fragment (peak D) from *S. solfataricus* P2 (KpnI) for cloning into pNER803. The plasmid was used in AFM experiments.
NER36	ATAATAAAGCTTAAGGAAGTAGAGTC	Forward primer to amplify a 1028 bp DNA fragment (containing peak D) from *S. solfataricus* P2 (HindIII) for cloning into pNER804. The plasmid was used in AFM experiments.
NER37	ATAATAGCATGCAGACTGGCTAAGAC	Reverse primer to amplify a 1028 bp DNA fragment (containing peak D) from *S. solfataricus* P2 (SphI) for cloning into pNER804. The plasmid was used in AFM experiments.

NER7	TTTAAATGTATATCCTCGC	Forward primer to amplify *S. solfataricus* P2 genomic region 20475-20674 (peak B4) for EMSA and DNaseI footprint
NER8	AGGGTAATGATATGGACTT	Reverse primer to amplify *S. solfataricus* P2 genomic region 20475-20674 (peak B4) for EMSA and DNaseI footprint
NER9	GATTTTTATGAATTAGGATTCA	Forward primer to amplify *S. solfataricus* P2 genomic region 22703-22902 (peak B5) for EMSA and DNaseI footprint
NER10	GCATCTATGGCATCTG	Reverse primer to amplify *S. solfataricus* P2 genomic region 22703-22902 (peak B5) for EMSA and DNaseI footprint
NER11	GCTCGTAATTGAGGC	Forward primer to amplify *S. solfataricus* P2 genomic region 24311-24510 (peak B6) for EMSA and DNaseI footprint
NER12	ACTTAACTCTAGATAATAAATC	Reverse primer to amplify *S. solfataricus* P2 genomic region 24311-24510 (peak B6) for EMSA and DNaseI footprint
NER47	CTCAGCTGTCGGTAATAC	Forward primer to amplify *S. solfataricus* P2 genomic region 18531-18730 (peak B3) for EMSA and DNaseI footprint
NER48	AGGAGTATGGAATACGTC	Reverse primer to amplify *S. solfataricus* P2 genomic region 18531-18730 (peak B3) for EMSA and DNaseI footprint
AK85-B-forw	GAATGTCACAAGGTTCTT	Forward primer to amplify *S. solfataricus* P2 genomic region 1001932-1002131 (peak D) for EMSA and DNaseI footprint

AK86-B-rev	AACACTCTAACTATTTATG	Reverse primer to amplify *S. solfataricus* P2 genomic region 1001932-1002131 (peak D) for EMSA and DNaseI footprint
NER42	GTATTTTTATTTTTGGATAA	Forward primer to amplify *S. acidocaldarius* genomic region 166146-166345 for DNaseI footprint
NER43	GGTTTAAAACCTATTCAC	Reverse primer to amplify *S. acidocaldarius* genomic region 166146-166345 for DNaseI footprint
Saci_0203_Forw	ATATATCATATGAGTGAGTTAGACTTAGAC	Forward primer to amplify *S. acidocaldarius segB* gene (*saci_0203*) for cloning into pET22b (NdeI)
Saci_0203_Back	ATATATCTCGAGACTCTTTTTACTCTCTAA	Reverse primer to amplify *S. acidocaldarius segB* gene (*saci_0203*) for cloning into pET22b (XhoI)

Supplementary Table 4. Constructs used in this study.

Plasmid	Description	Source/Reference
pET22b	Expression vector containing the T7 promoter and the sequence encoding a hexa-his-tag at the 3' of the cloned gene	Novagen
pET-*sso035*	Expression plasmid containing the *S. solfataricus segB* gene with no STOP codon cloned into NdeI and XhoI restriction sites	Kalliomaa-Sanford *et al* (2012)[4]
pET22b-*sso034*-stop	Expression plasmid containing the *S. solfataricus segA* gene with STOP codon cloned into NdeI and XhoI restriction sites	This work
pET22b-*saci0203*	Expression plasmid containing the *S. acidocaldarius segB* (*saci_0203*) gene with no STOP codon cloned into NdeI and XhoI restriction sites	This work
pET2268	Plasmid used for the construction of *S. solfataricus* Δ*segB* strain and containing *lacS* gene	Albers and Driessen (2008)[5]
pET2268-segB-KO-US-fragment	pET2268 containing the 823 bp region upstream of *S. solfataricus segB* cloned into KpnI and NcoI restriction sites upstream of the *lacS* gene	This work
pET2268-segB-KO-DS-fragment	pET2268 containing the 1150 bp region downstream of *S. solfataricus segB* cloned into BamHI and NotI restriction sites downstream of the *lacS* gene	This work
pSVA406	pGEM-T derived plasmid containing the *pyrEF* cassette from *S. solfataricus*, used for overlap extension PCR to construct *S. acidocaldarius* deletion strains	Wagner *et al* (2012)[6]
pSSR	Expression vector for *S. solfataricus*	Zheng *et al* (2012)[7]
p*segB*	pSSR containing *S. solfataricus segB* gene	This work
pUC18	Cloning vector	Norrander *et al* (1983)[8]
pNER803	pUC18 containing a 1028 bp *S. solfataricus* region that harbours peak D cloned into SphI and BamHI restriction sites	This work
pNER804	pNER803 containing a 126 bp *S. solfataricus* region that harbour the high-affinity SegB binding site located in peak D, cloned into BamHI and KpnI restriction sites	This work

pNER805	pNER804 containing a 1028 bp *S. solfataricus* region that harbours peak D cloned into SphI and HindIII restriction sites	This work

Supplementary references

1. Saha, C. K., Pires, R. Sanches Pires, R., Brolin, H., Delannoy, M. and Atkinson, G. C. FlaGs and webFlaGs: discovering novel biology through the analysis of gene neighbourhood conservation. *Bioinformatics* **37**, 1312-1314 (2021).

2. Huerta-Cepas, J., Serra, F. & Bork, P. ETE 3: reconstruction, analysis, and visualization of phylogenomic data. *Mol. Bio. Evol.* **33**, 1635–1638 (2016).

3. Hirabayashi, K., Yuda, E., Tanaka, N., Katayama, S., Iwasaki, K., Matsumoto, T., Kurisu, G., Outten, F.W., Fukuyama, K., Takahashi, Y. & Wada, K. Functional Dynamics Revealed by the Structure of the SufBCD Complex, a Novel ATP-binding Cassette (ABC) Protein That Serves as a Scaffold for Iron-Sulfur Cluster Biogenesis. *J. Biol. Chem.* **290**, 29717-29731 (2015).

4. Kalliomaa-Sanford, A. K., Rodriguez-Castañeda, F. A., McLeod, B. N., Latorre-Rosellò, Smith, J. H., Reimann, J., Albers, S. V. & Barillà, D. Chromosome segregation in archaea mediated by a hybrid DNA partition machine. *Proc. Natl. Acad. Sci. USA* **109**, 3754-3759 (2012).

5. Albers, S. V. & Driessen, A. J. (2008) Conditions for gene disruption by ho,ologous recombination of exogenous DNA into the *Sulfolobus solfataricus* genome. *Archaea* **2**, 145-149 (2008).

6. Wagner, M., van Wolferen, M., Wagner, A., Lassak, K., Meyer, B. H., Reimann, J. & Albers S. V. Versatile genetic tool box for the crenarchaeote *Sulfolobus acidocaldarius*. *Front. Microbiol.* **3**, 214 (2012).

7. Zheng, T., Huang, Q., Zhang, C., Ni, J., She, Q. & Shen, Y. Development of a simvastatin selection marker for a hyperthermophilic acidophile, *Sulfolobus islandicus*. *Appl. Environ. Microbiol.* **78**, 568-574 (2012).

8. Norrander, J., Kempe, T. and Messing, J. Construction of improved M13 vectors using oligodeoxynucleotide-directed mutagenesis. *Gene* **26**, 101-106 (1983).
