# Supplementary material for: Coupling chromosome organization to genome segregation in Archaea
Source: Nat Commun. 2025 Jul 22;16:6759. doi: 10.1038/s41467-025-61997-3 (PMC12284271; doi:10.1038/s41467-025-61997-3)
Supplement: Supplementary file 2 — Description of Additional Supplementary Files [file 41467_2025_61997_MOESM2_ESM.pdf]

Description of Additional Supplementary Files:

Supplementary Data 1: AlphaFold 2 predicted best model of *M. hakonensis* SegB monomer

Supplementary Data 2: AlphaFold 2 predicted best model of *M. sedula* SegB monomer

Supplementary Data 3: AlphaFold 2 predicted best model of *M. hakonensis* SegB dimer

Supplementary Data 4: AlphaFold 2 predicted model 5 for *M. hakonensis* dimer

Supplementary Data 5: AlphaFold 3 predicted best model for *M. hakonensis* dimer
