# Supplementary material for: Coupling chromosome organization to genome segregation in Archaea
Source: Nat Commun. 2025 Jul 22;16:6759. doi: 10.1038/s41467-025-61997-3 (PMC12284271; doi:10.1038/s41467-025-61997-3)
Supplement: Supplementary file 8 — Reporting Summary [file 41467_2025_61997_MOESM8_ESM.pdf]

nature portfolio

Corresponding author(s): Daniela Barilla`

Last updated by author(s): Jun 25, 2025

Reporting Summary

Nature Portfolio wishes to improve the reproducibility of the work that we publish. This form provides structure for consistency and transparency in reporting. For further information on Nature Portfolio policies, see our Editorial Policies and the Editorial Policy Checklist.

Statistics

For all statistical analyses, confirm that the following items are present in the figure legend, table legend, main text, or Methods section.

n/a	Confirmed	
☐	☒	The exact sample size (*n*) for each experimental group/condition, given as a discrete number and unit of measurement
☐	☒	A statement on whether measurements were taken from distinct samples or whether the same sample was measured repeatedly
☐	☒	The statistical test(s) used AND whether they are one- or two-sided<br>*Only common tests should be described solely by name; describe more complex techniques in the Methods section.*
☒	☐	A description of all covariates tested
☒	☐	A description of any assumptions or corrections, such as tests of normality and adjustment for multiple comparisons
☐	☒	A full description of the statistical parameters including central tendency (e.g. means) or other basic estimates (e.g. regression coefficient) AND variation (e.g. standard deviation) or associated estimates of uncertainty (e.g. confidence intervals)
☐	☒	For null hypothesis testing, the test statistic (e.g. *F*, *t*, *r*) with confidence intervals, effect sizes, degrees of freedom and *P* value noted<br>*Give P values as exact values whenever suitable.*
☒	☐	For Bayesian analysis, information on the choice of priors and Markov chain Monte Carlo settings
☒	☐	For hierarchical and complex designs, identification of the appropriate level for tests and full reporting of outcomes
☐	☒	Estimates of effect sizes (e.g. Cohen's *d*, Pearson's *r*), indicating how they were calculated

Our web collection on statistics for biologists contains articles on many of the points above.

Software and code

Policy information about availability of computer code

Data collection	CytExpert v. 2.3.0.84 (Beckman Coulter) was used for flow cytometry data acquisition; Image Lab v. 6.1 (Bio-Rad) was used for agarose and protein gels.
Data analysis	GraphPad Prism v. 10.3.0 and v. 10.4 (GraphPad Software) was used for statistical analyses.<br>NTanalysis v.1.0 (NanoTemper Technologies) was used to analyze protein-protein interactions.<br>FlowJo v. 10.10.0 (Becton, Dickinson, BD) was used to analyze flow cytometry data.<br>Fiji/ImageJ v. 2.14.0 (https://fiji.sc/) and Volocity v.5.5 (Perkin Elmer) were used to analyze microscopy images.<br>WebFlaGs (https://server.atkinson-lab.com/webflags) was used to investigate the genomic neighbours of segAB.<br>UCSF ChimeraX v. 1.8 and 1.9 was used to visualize the predicted structure of different proteins.<br>AlphaFold 2 (AF2) accessed through ColabFold (Mirdita, M. et al. Nat. Methods 19, 679-689, 2022). and AlphaFold 3 (AF3)(https://alphafoldserver.com/welcome) were used to obtain the predicted structure of different SegB homologues.<br>Dali server (http://ekhidna2.biocenter.helsinki.fi/dali/) and FoldSeek (https://search.foldseek.com/search) were used to find structural homologues of M. hakonensis SegB.<br>Gwyddion v. 2.57 (http://gwyddion.net) and WSxM v. 3.0 (Horcas, I. et al. Rev. Sci. Instrum. 78, 013705, 2007) were used to analyze AFM images.<br>MEME-ChIP v. 5.5.6 (https://meme-suite.org/meme/doc/meme-chip.html) and FIMO v. 5.5.6 (https://meme-suite.org/meme/doc/fimo.html) were used to identify SegB binding sites within the ChIP-seq peaks and to identify similar sites in the genome of different archaeal genera.<br>Sequence reads were mapped to the S. solfataricus P2 genome (NCBI Reference Sequence: NC_002754) with Bowtie 1.0.0-5 (Langmead et al 2009 PMID: 19261174).<br>Homer v4.9.1 was used for peak calling for S. sofataricus ChIP-seq samples

MACS2 program was employed for peak calling for S. acidocaldarius ChIP-seq samples, using the following command for example: macs2 callpeak -t ./WT/output.sorted.bam -c ./delta_segB_control/output.sorted.bam -f BAM -g 2e+6 --no_model.

Custom R scripts (https://github.com/TungLeLab/DSM639_Sacidocaldarius_ChIP_seq) were used to generate ChIP-seq profiles.
A custom MATALAB script was used to analyse immunofluorescence microscopy images (https://github.com/BarillaLab/Immunofluorescence_Analysis). All the codes are accessible without any restriction.

For manuscripts utilizing custom algorithms or software that are central to the research but not yet described in published literature, software must be made available to editors and reviewers. We strongly encourage code deposition in a community repository (e.g. GitHub). See the Nature Portfolio guidelines for submitting code & software for further information.

Data

Policy information about availability of data

All manuscripts must include a data availability statement. This statement should provide the following information, where applicable:

- Accession codes, unique identifiers, or web links for publicly available datasets
- A description of any restrictions on data availability
- For clinical datasets or third party data, please ensure that the statement adheres to our policy

All relevant data are provided within the manuscript, supplementary data and Souce Data files. ChIP-seq data have been uploaded to the GEO repository (accession codes GSE169604 and GSE297822). Data in the GEO are fully accessible to the public. PDB of predicted structural models are provided as Supplementary data files. The DOI for AF3-predicted M. hakonensis SegB structure is 10d08087466be71f. The codes can be accessed without restrictions.

Research involving human participants, their data, or biological material

Policy information about studies with human participants or human data. See also policy information about sex, gender (identity/presentation), and sexual orientation and race, ethnicity and racism.

Reporting on sex and gender	The study did not involve human participants or human material.
Reporting on race, ethnicity, or other socially relevant groupings	The study did not involve human participants or human material.
Population characteristics	The study did not involve human participants or human material.
Recruitment	The study did not involve human participants or human material.
Ethics oversight	The study did not involve human participants or human material.

Note that full information on the approval of the study protocol must also be provided in the manuscript.

Field-specific reporting

Please select the one below that is the best fit for your research. If you are not sure, read the appropriate sections before making your selection.

☒ Life sciences ☐ Behavioural & social sciences ☐ Ecological, evolutionary & environmental sciences

For a reference copy of the document with all sections, see nature.com/documents/nr-reporting-summary-flat.pdf

Life sciences study design

All studies must disclose on these points even when the disclosure is negative.

Sample size

No statistical method was used to predetermine sample size. All sample sizes were determined in accordance with published literature relevant to the particular type of experiment.

Data exclusions

No data exclusion was performed in this study.

Replication

Most of the experiments were performed in triplicates with three biological replicates in different days with similar results.

ChIP-seq experiments were done in duplicates, considering that the field accepted standard for the number of biological replicates for ChIP-seq is at least two.

Microscopy and AFM experiments were performed at least in triplicates producing similar results.

Flow cytometry experiments were performed in three biological replicates for WT and Delta_segB(psegB) and in two biological replicates for

	Delta_segB. The comparability of the results from all the biological replicates indicated the reproducibility of the findings.<br><br>MicroScale Thermophoresis (MST) experiments were performed in triplicates. The results from independent experiments were comparable.<br><br>DNaseI and EMSA experiments were performed at least in duplicates, generating comparable results.
Randomization	No randomization was applied to this study.
Blinding	Blinding was used for microscopy analysis of cell size of S. solfataricus and S. acidocaldarius samples.<br><br>Blinding was not necessary for the other experiments reported in this study, as ChIP-seq data were collected using an Illumina MiSeq or Hiseq system, flow cytometry data were collected using the CytoFlex LX (Beckman Coulter) and software CytExpert v. 2.3.0.84 and analysed using FlowJo v. 10.10.0. Blinding was not applicable to any biochemical assays performed in this study.

Reporting for specific materials, systems and methods

We require information from authors about some types of materials, experimental systems and methods used in many studies. Here, indicate whether each material, system or method listed is relevant to your study. If you are not sure if a list item applies to your research, read the appropriate section before selecting a response.

Materials & experimental systems

n/a	Involved in the study
☐	☒ Antibodies
☒	☐ Eukaryotic cell lines
☒	☐ Palaeontology and archaeology
☒	☐ Animals and other organisms
☒	☐ Clinical data
☒	☐ Dual use research of concern
☒	☐ Plants

Methods

n/a	Involved in the study
☐	☒ ChIP-seq
☐	☒ Flow cytometry
☒	☐ MRI-based neuroimaging

Antibodies

Antibodies used	Immunofluorescence microscopy<br>Primary antibodies used against S. solfataricus<br>1. Polyclonal anti-SegA (1:250) (raised against recombinant SegA by Genosphere)<br>2. Polyclonal anti-SegB (1:100) (raised against recombinant SegB by Genosphere)<br>Both antibodies were immunopurified by us using SegA- or SegB-coupled affinity chromatography columns.<br><br>Secondary antibody used against S. solfataricus<br>Goat anti-rabbit IgG conjugated to Alexa Fluor 555 (3:1000) (ThermoFisher, Invitrogen, cat. # A-21428).<br><br>ChIP-seq<br>S. solfataricus and S. acidocaldarius ChIP-seq<br>50 uL of immune-purified anti-SegB (5.7 mg/mL) were added to 50 uL of sonicated samples.
Validation	Sera and pre-immune sera were tested by Genosphere using ELISA.<br><br>Immuno-purified antibodies were validated by performing Western blots with purified proteins and cell extracts.

Plants

Seed stocks

The study did not involve plants.

Novel plant genotypes

The study did not involve plant.

Authentication

The study did not involve plants.

ChIP-seq

Data deposition

☒ Confirm that both raw and final processed data have been deposited in a public database such as GEO.

☒ Confirm that you have deposited or provided access to graph files (e.g. BED files) for the called peaks.

Data access links
May remain private before publication.

https://www.ncbi.nlm.nih.gov/geo/query/acc.cgi?acc=GSE169604

https://www.ncbi.nlm.nih.gov/geo/query/acc.cgi?acc=GSE297822

Files in database submission

S. solfataricus P2 and PBL2025 CHIP-seq samples

GSM5210601 DBLab_ChIP_seq_WT_anti_SegB_1
GSM5210602 DBLab_ChIP_seq_WT_anti_SegB_2
GSM5210603 DBLab_ChIP_seq_WT_anti_SegB_3
GSM5210604 DBLab_ChIP_seq_WT_anti_SegB_4
GSM5210605 DBLab_ChIP_seq_WT_anti_SegB_5
GSM5210606 DBLab_ChIP_seq_WT_anti_SegB_6
GSM5210607 DBLab_ChIP_seq_delta_anti_SegB_1
GSM5210608 DBLab_ChIP_seq_delta_anti_SegB_2

S. acidocadarius DSM 639 CHIP-seq samples

GSM9000400 S_acidocaldarius_delta_segB_ChIPseq_rep1
GSM9000401 S_acidocaldarius_delta_segB_ChIPseq_rep2
GSM9000402 S_acidocaldarius_WT_ChIPseq_rep1
GSM9000403 S_acidocaldarius_WT_ChIPseq_rep2

Genome browser session
(e.g. UCSC)

Not applicable because there is no UCSC browser for the reference genomes of S. sofataricus or S. acidocaldarius. However, all processed data have been uploaded to GEO and are available to the public, and actual ChIP-seq profiles are shown in the figures in the manuscript.

Methodology

Replicates

x2 biological replicates for each ChIP-seq experiment

Sequencing depth

1.3 to 2.3 million mappable reads for each ChIP-seq experiment (genome size is ~ 2Mb), single-end 50 nt reads

Antibodies

Anti-SegB rabbit polyclonal antibody (against S. solfataricus SegB) and anti-SegB rabbit polyclonal antibody (against S. acidocaldarius SegB).

Peak calling parameters

Homer v4.9.1 was used for peak calling for S. sofataricus ChIP-seq samples
MACS2 program was employed for peak calling for S. acidocaldarius ChIP-seq samples, using the following command for example:
macs2 callpeak -t ./WT/output.sorted.bam -c ./delta_segB_control/output.sorted.bam -f BAM -g 2e+6 --no_model

Data quality

1.3 to 2.3 million mappable reads for each ChIP-seq experiment (genome size is ~ 2Mb). We also only consider MACS2-called peaks with fold enrichment > 5 (i.e. log 2 enrichment > 2.3) and -log10(FDR q value) > 30. And peaks described in the manuscripts were also inspected visually using Artemis genome browser in both replicates (Supplementary Table S1 and S2), and the underlying SegB binding sequences were also highlighted underneath each peak (see Supplementary Table S1 and S2) .

Software

Sequence reads were mapped to the S. solfataricus P2 genome (NCBI Reference Sequence: NC_002754) with Bowtie 1.0.0-5 (Langmead et al 2009 PMID: 19261174) using the following command: bowtie -m 1 -n 1 --best --strata -p 4 --chunkmbs 512 NC_002754_bowtie --sam *.fastq > output.sam Samtools 0.1.19-1 and Bedtools 2.17.0-1 (Li et al 2009 PMID: 19505943, Quinland et al 2010 PMID: 20110278) were used to compute and extract the sequencing coverage using the following command: samtools faidx NC_002754.fna samtools import NC_002754.fna.fai *.sam output.bam samtools sort output.bam output.sorted samtools index output.sorted.bam bedtools genomecov -d -ibam output.sorted.bam -g NC_002754.fna > coverage_output.txt
Peak calling - Homer v4.9.1
Genome_build: NC_002754

For S. acidocaldarius ChIP-seq, a simialr mapping procedure was also employed but using Genome_build: NC_007181.1. And when necessary, MACS2 were employed to call peaks.

Finally, ChIP-seq profiles were plotted with the x-axis representing genomic positions and the y-axis is the number of reads per base pair per million mapped reads (RPBPM) or number of reads per kb per million mapped reads (RPKPM) using custom R scripts.

Flow Cytometry

Plots

Confirm that:

- [x] The axis labels state the marker and fluorochrome used (e.g. CD4-FITC).
- [x] The axis scales are clearly visible. Include numbers along axes only for bottom left plot of group (a 'group' is an analysis of identical markers).
- [x] All plots are contour plots with outliers or pseudocolor plots.
- [x] A numerical value for number of cells or percentage (with statistics) is provided.

Methodology

Sample preparation	S. solfataricus and S. acidocaldarius cells were grown at 75°C in Brock's medium, pH 3.5, (50 mL) plus supplements. One mL aliquots were collected from exponentially growing cultures (OD600 ~0.2). Cells were fixed with 70% ice-cold ethanol for a minimum of 30 minutes on ice and stored at 4°C until use. Fixed cells were harvested by centrifugation (8 minutes at 6000 g at 4°C) and washed once by resuspension in 1 mL of buffer (10 mM Tris-HCL, pH 7.5, 10 mM MgCl2). Cells were resuspended in 500 µL buffer and mixed with a freshly made 200-fold dilution of Quant-iT™PicoGreen® fluorescent reagent (Invitrogen™) to stain the DNA. Samples were incubated in the dark for 30 minutes. Flow cytometry experiments were performed using the CytoFLEX LX (Beckman Coulter) by exciting the PicoGreen-stained cells with the 488 nm laser.
Instrument	CytoFLEX LX (Beckman Coulter) equipped with 375, 405, 488, 561, 640 and 808 nm lasers.
Software	CytExpert v. 2.3.0.84 was used for data acquisition and FlowJo v. 10.10.0 was used for data analysis.
Cell population abundance	No cell sorting was performed.
Gating strategy	A threshold was set at 2366 height on side scatter. A gate was set to select single cells and to remove debris present in the bottom corner of the dot plots. Supplementary Figure 6c provides examples of the gating strategy.

- [x] Tick this box to confirm that a figure exemplifying the gating strategy is provided in the Supplementary Information.
